# Dissociating Representations of Time and Number in Reinforcement-Rate Learning by Deletion of the GluA1 AMPA Receptor Subunit in Mice

**DOI:** 10.1177/0956797620960392

**Published:** 2021-01-04

**Authors:** Joseph M. Austen, Corran Pickering, Rolf Sprengel, David J. Sanderson

**Affiliations:** 1 Department of Psychology, Durham University; 2 Max Planck Institute for Medical Research, Institute for Anatomy and Cell Biology, Heidelberg University

**Keywords:** learning, rewards, time estimation, open data

## Abstract

Theories of learning differ in whether they assume that learning reflects the strength of an association between memories or symbolic encoding of the statistical properties of events. We provide novel evidence for symbolic encoding of informational variables by demonstrating that sensitivity to time and number in learning is dissociable. Whereas responding in normal mice was dependent on reinforcement rate, responding in mice that lacked the GluA1 AMPA receptor subunit was insensitive to reinforcement rate and, instead, dependent on the number of times a cue had been paired with reinforcement. This suggests that GluA1 is necessary for weighting numeric information by temporal information in order to calculate reinforcement rate. Sample sizes per genotype varied between seven and 23 across six experiments and consisted of both male and female mice. The results provide evidence for explicit encoding of variables by animals rather than implicit encoding via variations in associative strength.

Animals are highly sensitive to the temporal properties of predictive cues. For example, long-duration cues elicit weaker conditioned responding than short-duration cues (*cue-duration effect*; Harris & Carpenter, 2011; Harris, Patterson, & Gharaei, 2015). There has been a divergence in the theoretical explanations for this cue-duration effect. Decision-making theories account for the cue-duration effect by assuming that animals symbolically encode temporal durations by use of an internal clock in order to extract statistical information about events, such as the reinforcement rate during a cue’s presentation (Balsam & Gallistel, 2009; Gallistel & Gibbon, 2000). Encoding of rate information allows animals to behave optimally in an efficient manner by responding at a low rate for cues with a low reinforcement rate and a high rate for cues with a high reinforcement rate. For example, in choice behavior, it is often found that the response rate to one behavioral option relative to other behavioral options matches the relative reinforcement rate of that option (Herrnstein, 1961). Therefore, animals respond at a low rate to cues of long durations not because they are learned poorly but because they signal a low rate of reinforcement.

In contrast, associative-learning theories assume that the strength of conditioned responding elicited by a cue reflects the strength of an association between the cue and an outcome (e.g., a tone and food reward). Therefore, long-duration cues elicit weaker conditioned responding than short-duration cues because the strength of associative learning is weaker than for short-duration cues. Whereas associative strength may be affected by the statistical properties of cues, the associative strength of a cue is not a representation of the statistical properties of cues. Thus, models of associative learning assume that associative strength is blind to the circumstances that led to its acquisition.

The associative theory of Wagner (1981) proposes that temporal sensitivity is a by-product of time-dependent changes in stimulus processing that impact associative learning. Thus, short-term habituation throughout the presentation of a cue results in a loss of attention that limits the amount of learning that can occur when reinforcement is presented (Best & Gemberling, 1977; Bouton & Sunsay, 2003). This mechanism results in learning being sensitive to the temporal properties of cues, but animals have not explicitly encoded time and number information in order to calculate reinforcement rate.

To identify the psychological mechanisms underlying sensitivity to temporal information, we began by testing the simple short-term-habituation account in mice with a knockout of *Gria1* (*Gria1*^–/–^), the gene that encodes for the GluA1 subunit of the AMPA receptor for glutamate (Zamanillo et al., 1999). GluA1 is necessary for stimulus-specific short-term habituation (Sanderson et al., 2009; Sanderson et al., 2011) but not long-term memory (Reisel et al., 2002; Sanderson et al., 2009; Schmitt, Deacon, Seeburg, Rawlins, & Bannerman, 2003) and, therefore, plays a specific role in short-term memory. If the cue-duration effect is caused by short-term habituation during the presentation of a cue, then GluA1 deletion should impair the cue-duration effect and increase learning with the long-duration cue. Although enhanced learning of the long-duration cue in *Gria1*^–/–^ mice would support the short-term-habituation account, an alternative explanation may be that GluA1 regulates the calculation of reinforcement rate. Tests of this alternative hypothesis were conducted in subsequent experiments to dissociate associative- and symbolic-encoding accounts of the role of GluA1 in reinforcement-rate learning.

## Experiments 1 to 4

In Experiment 1, we tested the effect of GluA1 deletion on the cue-duration effect. In Experiments 2 to 4, we examined the role of reinforcement rate and frequency of reinforcement in determining the role of GluA1 in the cue-duration effect observed in Experiment 1.

### Method

#### Participants

Mice were *Gria1*^–/–^ and wild-type age-matched littermates bred in the Life Sciences Support Unit at Durham University (for details of genetic construction, breeding, and subsequent genotyping, see Zamanillo et al., 1999). The mice were originally derived from the 129S2svHsd and C57BL/6J/OlaHsd strains and have been subsequently backcrossed onto the C57BL/6J line. Mice were housed in groups of one to 12 in a temperature-controlled holding room on a 12-hr light-dark cycle (light period: 8 a.m. to 8 p.m.). For several days prior to the start of testing, the weights of the mice were reduced by restricting access to food, and they were maintained at 85% of their free-feeding weights throughout the experiment. Mice had ad libitum access to water in their home cages. All procedures were in accordance with the United Kingdom Animals (Scientific Procedures) Act (1986) under Project License Number PPL 70/7785.

**Statement of Relevance**

Learning is fundamental to behavioral adaptation in humans as well as nonhuman animals. Some of our strongest evidence for how learning occurs comes from animal models in which candidate mechanisms are altered by genetic manipulations. We conducted research with these animals to solve a fundamental question of learning: whether animals encode the statistical properties of events (e.g., duration and number) or whether the statistical properties of events simply affect the strength of associations between them. We used mice that lacked a receptor necessary for the time-sensitive calculations required for rate sensitivity. Across multiple experiments, learning in these “knockout” mice was dependent on the number of cue-reinforcement pairings. Because the knockout switched learning from being sensitive to rate to being sensitive to number, we concluded that learning reflects encoding of statistical information. The challenge now is to determine the precise mechanisms by which animals represent the quantitative variables required for this type of learning.

#### Apparatus

A set of eight identical operant chambers (interior dimensions: 15.9 × 14.0 × 12.7 cm; ENV-307A; all equipment was obtained from Med Associates, St. Albans, VT), enclosed in sound-attenuating cubicles (ENV-022V), were used. The operant chambers were controlled by Med-PC IV software (SOF-735; Med Associates, 2013). The side walls were made from aluminum, and the front and back walls and the ceiling were made from clear Perspex. The chamber floors each comprised a grid of stainless-steel rods (diameter = 0.32 cm) spaced 0.79 cm apart and running perpendicular to the front of the chamber (ENV-307A-GFW). A food magazine (2.9 × 2.5 × 1.9 cm; ENV-303M) was situated in the center of one of the sidewalls of the chamber, into which sucrose pellets (14 mg; TestDiet, St. Louis, MO) could be delivered from a pellet dispenser (ENV-203-14P). An infrared beam (ENV-303HDA) across the entrance of the magazine recorded head entries at a resolution of 0.1 s. A fan (ENV-025F) was located within each of the sound-attenuating cubicles and was turned on during sessions, providing a background sound level of approximately 65 dB. Auditory stimuli were provided by a white-noise generator (ENV-325SM) that outputted a flat frequency response from 10 to 25,000 Hz at 80 dB, a clicker (ENV-335M) that operated at a frequency of 4 Hz at 80 dB, and a pure-tone generator (ENV-323AM) that produced a 2,900-Hz tone at 80 dB. Visual stimuli were a 2.8-W house light (ENV-315M) and two LEDs (ENV-321M) that were flashed (1 s on, 1 s off) alternating between left and right.

#### Procedure

Mice received one session of training per day with two 10-s cues and two 40-s cues. One cue of each duration was reinforced (on either 100% or 25% of trials, depending on the experiment) by presentation of a sucrose pellet at the termination of the cue. The remaining cues were not reinforced. Trials were separated by a fixed interval of 120 s (cue offset to cue onset). For approximately half of the mice within each genotype and sex, the 10-s cues were visual (house light, LEDs) and the 40-s cues were auditory (white noise, clicker). Within modality, the stimulus identity of reinforced and nonreinforced cues was counterbalanced as far as possible. For the remaining mice, the 10-s cues were auditory and the 40-s cues were visual, and the reinforcement contingencies within modality were similarly counterbalanced. The designs of Experiments 1 to 4 are summarized in Table 1.

**Table 1.** Designs of Experiments 1 to 6

Experiment and cue	Probability of reinforcement per trial	Reinforcement rate	Number of trials per block	Number of reinforcements per block
Exp. 1				
10 s	100%	Once every 10 s	6	6
40 s	100%	Once every 40 s	6	6
Exp. 2				
10 s	25%	Once every 40 s	6	~1.5
40 s	100%	Once every 40 s	6	6
Exp. 3				
10 s	25%	Once every 40 s	24	6
40 s	100%	Once every 40 s	6	6
Exp. 4				
10 s	100%	Once every 10 s	24	24
40 s	100%	Once every 40 s	6	6
Exp. 5				
10 s 100%	100%	Once every 10 s	6	6
10 s 25%	25%	Once every 40 s	24	6
Exp. 6				
10 s HRN	25%	Once every 40 s	24	6
10 s LRN	25%	Once every 40 s	6	~1.5

Note: Experiments 1 to 4 used cues that differed in duration (10-s and 40-s cues). Experiment 5 used cues that were matched for duration (10 s) but differed in probability of reinforcement per trial (100% and 25%). Experiment 6 used cues that were matched for duration and probability of reinforcement per trial but differed in number of reinforcements (high reinforcement number [HRN] and low reinforcement number [LRN]).

##### Experiment 1: different reinforcement rate, matched reinforcement number

Thirteen *Gria1*^–/–^ (7 female, 6 male) and 14 wild-type (6 female, 8 male) mice (free-feeding weights: 14.6–28.1 g) received 12 sessions of training in which the 10-s and 40-s reinforced cues were reinforced on every trial. Sessions consisted of six trials of each trial type (10 s reinforced, 10 s nonreinforced, 40 s reinforced, and 40 s nonreinforced). The cues were presented in a random order with the constraint that there was an equal number of each cue type every eight trials.

##### Experiment 2: matched reinforcement rate, different reinforcement number

Sixteen *Gria1*^–/–^ (9 female, 7 male) and 16 wild-type (9 female, 7 male) mice (free-feeding weights: 16.2–33.2 g) received 12 sessions of training in which the 10-s reinforced cue was reinforced on only 25% of trials and the 40-s reinforced cue was reinforced on every trial. Sessions consisted of six trials of each trial type (10 s reinforced, 10 s nonreinforced, 40 s reinforced, and 40 s nonreinforced). The partially reinforced 10-s cue was reinforced either once or twice per session, and it was ensured that across blocks of two sessions, the 10-s cue was reinforced three times. The cues were presented in a random order with the constraint that there was an equal number of each cue type every eight trials.

##### Experiment 3: matched reinforcement rate, matched reinforcement number

Seven *Gria1*^–/–^ (3 female, 4 male) and 10 wild-type (8 female, 2 male) mice (free-feeding weights: 16.4–29.7 g) received 24 sessions of training in which the 10-s reinforced cue was reinforced on only 25% of trials and the 40-s reinforced cue was reinforced on every trial. Sessions consisted of 12 trials of each of the 10-s cues (reinforced and nonreinforced) and three trials of each of the 40-s cues (reinforced and nonreinforced). The cues were presented in a random order with the constraint that there were four trials of each 10-s cue and one trial of each 40-s cue every 10 trials. Therefore, every 10 trials, the 10-s reinforced cue was reinforced once.

##### Experiment 4: different reinforcement rate, different reinforcement number

Eight *Gria1*^–/–^ (3 female, 5 male) and 10 wild-type (7 female, 3 male) mice (free-feeding weights: 15.9–33.0 g) received 24 sessions of training in which the 10-s and 40-s reinforced cues were reinforced on every trial. Sessions consisted of 12 trials of each of the 10-s cues (reinforced and nonreinforced) and three trials of each of the 40-s cues (reinforced and nonreinforced). The cues were presented in a random order with the constraint that there were four trials of each 10-s cue and one trial of each 40-s cue every 10 trials.

#### Data and statistical analysis

The number of head entries made to the food magazine during the presentation of each conditioned stimulus was recorded. For the main analysis of responding, the rate of head entries during nonreinforced cues was subtracted from the rate of head entries during reinforced cues that were matched for modality (difference score). Additional separate analyses were carried out on the rates of responding to the reinforced and nonreinforced cues in order to determine whether effects observed with difference scores were due to differences in responding to reinforced cues, nonreinforced cues, or both (see Fig. S5 in the Supplemental Material available online).

Additional analyses were conducted to dissociate learning from performance accounts of the results. For each experiment, the manner in which each cue was presented differed in terms of the frequency or cumulative duration of the cues. Whereas these manipulations may affect learning, they may also affect performance of responding. Performance effects can be ruled out by matching the test conditions for each cue (e.g., Holland, 2000). To assess responding under matched test conditions, we restricted analysis of the raw rates of responding to the reinforced cues to the first trial of each trial type per session. Therefore, responding on these trials reflects learning from prior experience rather than the influence of the particular manipulation (e.g., frequency or cumulative duration of presentation) on performance of responding. For both the 10-s and 40-s cues, rates of responding were for the whole duration of the cues rather than restricted to a particular duration or proportion of the cues in order to mitigate the influence of timing within the trial on response rates.

All data were analyzed using multifactorial analyses of variance (ANOVA) conducted in SPSS (Version 22). Counterbalancing of modality was included as a nuisance factor (because of mice responding more to auditory than visual cues). Interactions were analyzed with simple main-effects analysis or separate repeated measures ANOVA for within-subjects factors with more than two levels. Where sphericity of within-subjects variables could not be assumed, a Greenhouse-Geisser correction was applied to produce more conservative *p* values. Where the correction was applied, the corrected degrees of freedom are reported. Null effects of genotype for the comparison of the effect of cue were further analyzed by calculating Bayes factors (BFs) to determine the relative size of evidence for the null result compared with the alternative hypothesis. Bayesian analyses were conducted in JASP (Version 0.9.2; JASP Team, 2020) using default priors. For interactions of factors, the reported BF compares models containing the effect of interest with equivalent models stripped of the effect, excluding higher order interactions. This method was suggested by Mathôt (2017). Within JASP, this was achieved by conducting a Bayesian repeated measures ANOVA and outputting effects across matched models.

On the basis of previous experiments (e.g., Austen & Sanderson, 2019) in which we manipulated cue duration in a similar manner as in Experiment 1, we estimated the cue-duration effect size (η_*p*_^2^) to be .75. Using G*Power (Faul, Erdfelder, Buchner, & Lang, 2009), we estimated group sizes of eight to be sufficient for detecting a significant effect of cue duration with power of .8 and an alpha of .05. Given the unknown effect of genotype on sensitivity to cue duration and manipulation of reinforcement rate, we chose to use samples per genotype in excess of eight when possible. The differences in sample sizes across experiments reflect the fluctuations in the success of the breeding colony at the time of running a particular experiment.

### Results

To create a measure of responding that indicated performance above baseline, we converted response rates to difference scores for the main analyses of training across blocks of trials. These difference scores were calculated by subtracting response rates for nonreinforced cues from response rates for reinforced cues that were matched for cue duration (e.g., response rate for 40-s reinforced cue minus response rate for 40-s nonreinforced cue). In Experiments 1 to 4, results were similar to those of previous work (Sanderson et al., 2017) in that *Gria1*^–/–^ mice responded at a significantly higher rate than wild-type mice to nonreinforced cues (*p*s ≤ .004; see Fig. S5). For each genotype, the effect of cue found with difference scores in Experiments 1 to 4 was also found with the raw rates of responding to reinforced cues (see Fig. S5), suggesting that the results observed with the difference scores were not simply a consequence of an effect of cue on responding to nonreinforced cues.

**Fig. 1.**
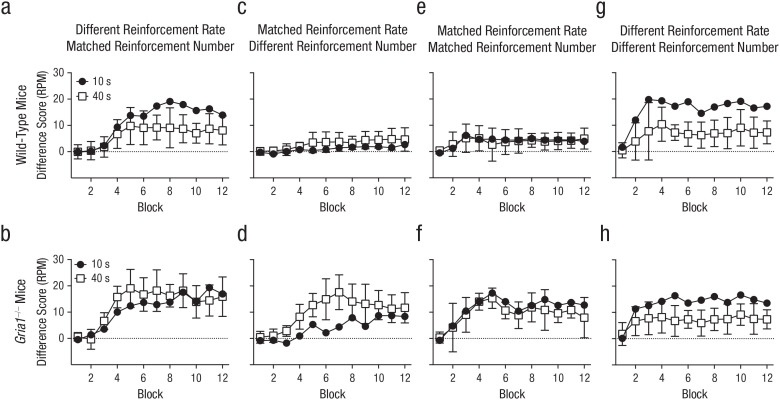
Difference scores in Experiments 1 to 4, separately for wild-type mice (top row) and *Gria1*^–/–^ mice (bottom row). Difference scores were calculated by subtracting mean response rate (number of food-magazine entries) to nonreinforced cues from mean response rate to reinforced cues (responses per minute [RPM]), matched for cue duration and frequency of presentation. Each graph shows scores for the two cue types across the 12 blocks in a session. For Experiments 1 and 2 (a–d), there were six 10-s trials and six 40-s trials per block. For Experiments 3 and 4 (e–h), there were twenty-four 10-s trials per block and six 40-s trials per block. In Experiment 1 (a, b), cues differed in reinforcement rate but not number of reinforcements. In Experiment 2 (c, d), cues were matched for reinforcement rate but not number of reinforcements. In Experiment 3 (e, f), cues were matched for reinforcement rate and number of reinforcements. In Experiment 4 (g, h), cues differed in reinforcement rate and number of reinforcements. Error bars on the white squares indicate 95% confidence intervals for the mean difference between the two cues.

In Experiment 1, we tested the effect of GluA1 deletion on the cue-duration effect by presenting mice with a short-duration, 10-s cue (e.g., light) and a long-duration, 40-s cue (e.g., noise) that both terminated with the presentation of a sucrose pellet (the two cues were presented on separate trials; see Table 1). Normal, wild-type mice showed the cue-duration effect: Difference scores were greater for short- than long-duration cues (see Fig. 1a). Consistent with the short-term-habituation account of the cue-duration effect, results revealed that *Gria1*^–/–^ mice did not exhibit the cue-duration effect, and the difference scores for the short- and long-duration cues were similar (see Fig. 1b). There was a significant Cue Duration × Genotype × Block interaction, *F*(5.32, 122.31) = 2.66, *p* = .023, η_*p*_^2^ = .11, 90% confidence interval (CI) = [.008, .16]. The significant three-way interaction among cue duration, block, and genotype was analyzed in separate ANOVAs for each genotype. For *Gria1*^−/−^ mice, there was a significant main effect of block, *F*(4.09, 44.95) = 20.7, *p* < .001, but no significant main effect of cue duration, *F*(1, 11) = 3.08, *p* = .11, and no Cue Duration × Block interaction, *F*(4.71, 51.79) = 2.32, *p* = .059. In contrast, wild-type mice showed significant main effects of cue duration, *F*(1, 12) = 12.4, *p* = .004, and block, *F*(2.43, 29.18) = 18.5, *p* < .001, and a significant Cue Duration × Block interaction, *F*(3.50, 42.04) = 3.27, *p* = .025. Further analysis of this interaction showed a significant effect of cue duration on Blocks 7 through 12 (*F*s > 5.3, *p*s < .04).

It is possible that the reduction in difference scores for the 40-s cue compared with the 10-s cue in wild-type mice may have been due to wild-type mice limiting their responding to the latter parts of the 40-s cue over the course of training (i.e., an inhibition-of-delay effect; Pavlov, 1927). If this is the case, then GluA1 deletion may have abolished the cue-duration effect by impairing inhibition of delay. An analysis of response rates during consecutive 10-s epochs of the 40-s cue over the course of training showed that mice tended to withhold responding during the first 10 s of the cue, but response rates were fairly constant in the last 30 s of the cue (see Fig. S7 in the Supplemental Material). This was true for both genotypes (see “Analysis of the Temporal Distribution of Responding for the 40 s Cue in Experiment 1” in the Supplemental Material), suggesting that the absence of a cue-duration effect in *Gria1*^–/–^ mice was not due to impaired inhibition of delay. In addition, wild-type mice showed significantly lower rates of responding in the last 10 s of the 40-s cue compared with the rate of responding across the whole duration of the 10-s reinforced cue, providing further evidence against an inhibition-of-delay account. This effect was absent in *Gria1*^–/–^ mice (see Fig. S7).

**Fig. 2.**
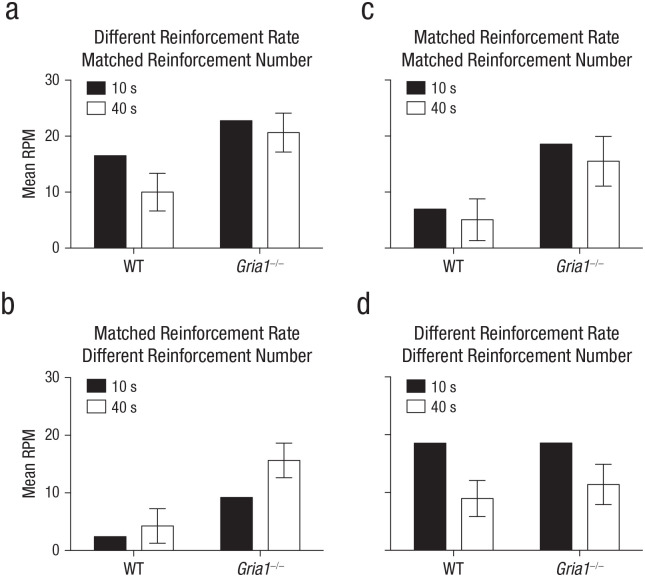
Rates of responding under matched test conditions in Experiments 1 to 4 (a–d, respectively). Each graph shows the mean rate of responding to reinforced cues (number of responses per minute [RPM]) in the first trial of a session, separately for 10-s and 40-s trials and for wild-type (WT) and *Gria1*^–/–^ mice. Response rates are collapsed across sessions. Error bars indicate 95% confidence intervals for the mean difference between the two cues.

To test whether cue had an effect on learning rather than performance of conditioned responding, we analyzed responding during the first trial of each trial type in each session (see Fig. 2a). There was a significant Cue × Genotype interaction, *F*(1, 23) = 4.56, *p* = .04, η_*p*_^2^ = .17, 90% CI = [.003, .37]. Simple main-effects analysis of the significant interaction revealed that wild-type mice responded significantly more to the 10-s cue than the 40-s cue, *F*(1, 23) = 16.18, *p* = .001, but *Gria1*^–/–^ mice did not (*F* < 1, *p* = .38). *Gria1*^–/–^ mice responded significantly more than wild-type mice to both the 10-s and 40-s cues, smallest *F*(1, 23) = 4.87, *p* = .04.

Although the results are consistent with the short-term-habituation account of the cue-duration effect, it is possible that, instead, GluA1 deletion abolished the cue-duration effect by impairing calculation of the reinforcement rate so that responding to the different duration cues was similar. In Experiment 2, to test the precise role of GluA1, we first tested whether the cue-duration effect reflects differences in the cumulative reinforcement rate. Naive mice were trained with short- and long-duration cues, but now the short-duration cue was reinforced on one in four trials, so that the cumulative rate of reinforcement across the short- and long-duration cues was matched (once every 40 s; see Table 1). Therefore, if the cue-duration effect was still evident despite the reinforcement rate being matched across cues, then this would provide evidence against the role of reinforcement rate in the cue-duration effect.

Consistent with reinforcement rate being a key determinant of responding, results showed that the cue-duration effect was now abolished in wild-type mice: Difference scores for the short- and long-duration cues were similar (see Fig. 1c). Difference scores for *Gria1*^–/–^ mice, however, were now greater for the long-duration cue compared with the short-duration cue (see Fig. 1d). There was a significant Cue Duration × Genotype × Block interaction, *F*(4.45, 124.50) = 2.87, *p* = .022, η_*p*_^2^ = .09, 90% CI = [.008, .15]. The significant three-way interaction among cue duration, block, and genotype was analyzed in separate ANOVAs for each genotype. For *Gria1*^−/−^ mice, there were significant main effects of cue duration, *F*(1, 14) = 12.2, *p* = .004, and block, *F*(4.39, 61.47) = 21.0, *p* < .001, and a significant Cue Duration × Block interaction, *F*(4.06, 56.80) = 4.37, *p* = .004. Further analysis of this interaction showed an effect of cue duration on Blocks 3 through 7 and 9 (*F*s > 6.0, *p*s < .03). For wild-type mice, there was a significant main effect of block, *F*(3.34, 46.69) = 7.75, *p* < .001, but no significant main effect of cue duration, *F*(1, 14) = 2.81, *p* = .12, and no Cue Duration × Block interaction, *F*(2.67, 37.34) = 1.11, *p* = .35.

To test whether cue had an effect on learning rather than performance of conditioned responding, we analyzed responding during the first trial of each trial type in each session (see Fig. 2b). There was a significant Cue × Genotype interaction, *F*(1, 28) = 4.91, *p* = .04, η_*p*_^2^ = .15, 90% CI = [.006, .34]. Simple main-effects analysis of the significant interaction revealed that *Gria1*^–/–^ mice responded significantly more to the 40-s cue than the 10-s cue, *F*(1, 28) = 19.50, *p* < .001, but wild-type mice did not, *F*(1, 28) = 1.64, *p* = .21. *Gria1*^–/–^ mice responded significantly more than wild-type mice to both the 10-s and 40-s cues, smallest *F*(1, 28) = 27.04, *p* < .001.

The absence of the cue-duration effect in wild-type mice in Experiment 2 may suggest that the cue-duration effect is caused by sensitivity to the reinforcement rate, but an alternative explanation is that the reduction in the difference scores for the short-duration cue was due to the fourfold reduction in the number of pairings with reinforcement rather than the reduction in reinforcement rate. Therefore, in Experiment 3, to test whether the learning with the short-duration cue was affected by reinforcement rate rather than simply the number of reinforcements, we repeated the procedure in naive mice, except that the partially reinforced short cue was now presented four times as often as the long-duration cue, so that the short- and long-duration cues were matched for both reinforcement rate and number of reinforcements (see Table 1).

The increase in number of reinforcements failed to increase difference scores for the short-duration cue compared with the long-duration cue in wild-type mice (see Fig. 1e), demonstrating that responding in wild-type mice is primarily sensitive to reinforcement rate. In contrast, the increase in the number of reinforcements resulted in difference scores for the short-duration cue now no longer differing from the long-duration cue in *Gria1*^–/–^ mice, and, therefore, both genotypes failed to show an effect of cue (see Fig. 1f). There were significant main effects of block, *F*(3.18, 41.37) = 11.4, *p* < .001, and genotype, *F*(1, 13) = 15.9, *p* = .002, and a significant Block × Genotype interaction, *F*(3.18, 41.37) = 3.70, *p* = .017. All other main effects and interactions were nonsignificant (*F*s < 1.0, *p*s > .40). Further analysis of the significant Block × Genotype interaction showed a significant effect of genotype on Blocks 4 through 12 (*F*s > 5.0, *p*s < .05).

To test whether cue had an effect on learning rather than performance of conditioned responding, we analyzed responding during the first trial of each trial type in each session (see Fig. 2c). The effect of cue was not significant, *F*(1, 13) = 1.03, *p* = .33. *Gria1*^–/–^ mice responded significantly more than wild-type mice, *F*(1, 13) = 27.22, *p* < .001. There was no significant interaction of factors (*F* < 1, *p* = .57).

These collective results suggest that *Gria1*^–/–^ mice are insensitive to reinforcement rate but are instead sensitive to the number of reinforcements: Difference scores were greater for the long-duration cue when it was reinforced more often than the short-duration cue (see Fig. 1d) but not when they were reinforced equally often (see Figs. 1b and 1f). If GluA1 deletion spares sensitivity to the number of pairings with reinforcement, then difference scores of *Gria1*^–/–^ mice should be greater for the short cue compared with the long cue if the short cue is reinforced more often than the long cue. This was tested in Experiment 4 by training naive mice with short- and long-duration cues. Each trial was reinforced, but the short cue was presented four times as often as the long cue (see Table 1). Consequently, the cues differed in reinforcement rate and number of reinforcements, but the cumulative exposure to each cue was matched. As predicted, difference scores for both *Gria1*^–/–^ and wild-type mice were greater with the short cue compared with the long cue (see Figs. 1g and 1h). There was a significant effect of cue, *F*(1, 14) = 71.1, *p* < .001, η_*p*_^2^ = .84, 90% CI = [.63, .89], and block, *F*(3.26, 45.57) = 12.5, *p* < .001. All other main effects and interactions were nonsignificant (*F*s < 2.1, *p*s > .11).

To test whether cue had an effect on learning rather than performance of conditioned responding, we analyzed responding during the first trial of each trial type in each session (see Fig. 2d). There was a significant effect of cue, *F*(1, 14) = 45.14, *p* < .001, η_*p*_^2^ = .76, 90% CI = [.50, .84]. There was no significant effect of genotype (*F* < 1, *p* = .58) and no significant interaction of factors (*F* < 1, *p* = .92).

## Experiments 5 and 6

Experiments 1 to 4 suggest that GluA1 plays a role in reinforcement-rate calculation independent of its role in short-term habituation. However, reinforcement rate was equated between cues by confounding the probability of reinforcement per trial with the duration of the cue (i.e., a 10-s cue that was reinforced on 25% of trials was compared with a 40-s cue that was reinforced on 100% of trials). Therefore, rate sensitivity may reflect an interaction between the effects of short-term habituation and partial reinforcement rather than rate calculation. If GluA1 is necessary for reinforcement-rate calculation, then GluA1 will be necessary for sensitivity to reinforcement rate even when cues are the same duration and, therefore, short-term habituation will equally affect learning with both cues. This prediction was tested in Experiment 5 by manipulating reinforcement rate between cues that were matched for duration. Experiment 6 tested whether GluA1 deletion spares sensitivity to the number of pairings with reinforcement when cues are matched for duration.

### Method

#### Experiment 5: different reinforcement rate, matched reinforcement number

Thirteen *Gria1*^–/–^ (7 female, 6 male) and 11 wild-type (5 female, 6 male) mice (free-feeding weights: 18.2–34.1 g) received 24 sessions of training, one per day, in which two 10-s cues (12 trials each per session) were presented four times as often as two other 10-s cues (three trials each per session). Trials were separated by a fixed interval of 120 s (cue offset to cue onset). One of the more frequently presented cues was reinforced (by presentation of a sucrose pellet at the termination of the cue) on a random 25% of trials (10 s 25%, reinforcement rate = once every 40 s), and the other was nonreinforced. One of the less frequently presented cues was reinforced on every trial (10 s 100%, reinforcement rate = once every 10 s) and the other was nonreinforced. For approximately half of the mice within each genotype and sex, the more frequently presented cues were visual (house light, LEDs) and the less frequently presented cues were auditory (white noise, clicker). Within modality, the allocation of reinforced and nonreinforced cues was counterbalanced as far as possible. For the remaining mice, the more frequently presented cues were auditory and the less frequently presented cues were visual, and the reinforcement contingencies within modality were similarly counterbalanced. The cues were presented in a random order with the constraint that there were four trials of each of the more frequently presented 10-s cues and one trial of each of the less frequently presented cues every 10 trials. Therefore, every 10 trials, the 10-s reinforced cue was reinforced once.

#### Experiment 6: matched reinforcement rate, different reinforcement number

Twelve *Gria1*^–/–^ (6 female, 6 male) and 12 wild-type (4 female, 8 male) mice (free-feeding weights: 17.3–33.8 g) received training that was the same as in Experiment 5 when cue durations were matched, except that the less frequently presented reinforced cue was reinforced on a random 25% of trials. Therefore, both the more frequent (high reinforcement number) and less frequent (low reinforcement number) reinforced cues were partially reinforced (25%). The low-reinforcement-number cue was reinforced on a random 25% of trials with a maximum of one trial out of three being reinforced per session and a minimum of none of the three trials being reinforced per session. Therefore, on any given session, the chance of a mouse receiving reinforcement on one of the three trials was 75% and on none of the three trials was 25%.

### Results

As in Experiments 1 to 4, we created a measure of responding that indicated performance above baseline by converting response rates to difference scores for the main analyses of training across blocks of trials. These difference scores were calculated by subtracting response rates for nonreinforced cues from response rates for reinforced cues that were matched for frequency of presentation. *Gria1*^–/–^ mice responded at a significantly higher rate than wild-type mice for nonreinforced cues in both Experiments 5 and 6 (*p*s ≤ .038; see Fig. S6 in the Supplemental Material). For each genotype, the effect of cue found with difference scores in Experiments 5 and 6 was also found with the raw rates of responding to reinforced cues (see Fig. S6), suggesting that the results observed with the difference scores were not simply a consequence of an effect of cue on responding to nonreinforced cues.

**Fig. 3.**
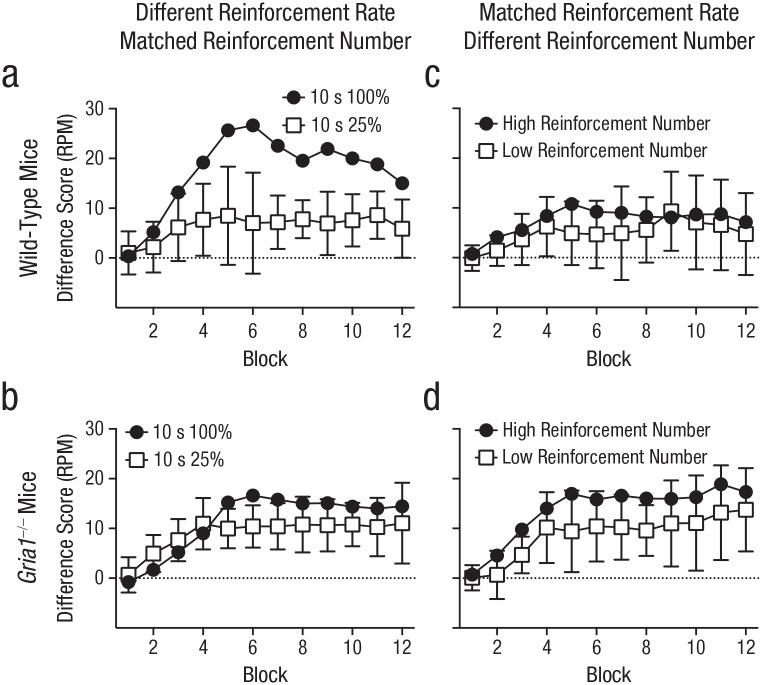
Difference scores in Experiments 5 and 6, separately for wild-type mice (top row) and *Gria1*^–/–^ mice (bottom row). Difference scores were calculated by subtracting mean response rate (number of food-magazine entries) to nonreinforced cues from mean response rate to reinforced cues (responses per minute [RPM]), matched for frequency of presentation. Each graph shows scores for the two cue types across 12 blocks of training. For Experiment 5, each block contained six 10-s cues that were reinforced on 100% of trials and twenty-four 10-s cues that were reinforced on 25% of trials. For Experiment 6, each block contained 24 high-reinforcement-number trials and six low-reinforcement-number trials. In Experiment 5 (a, b), cues differed in reinforcement rate but not number of reinforcements. In Experiment 6 (c, d), cues were matched for reinforcement rate but not number of reinforcements. Error bars on the white squares indicate 95% confidence intervals for the mean difference between the two cues.

Experiment 5 tested the prediction that GluA1 deletion impairs sensitivity to reinforcement rate when cues are matched for duration. Mice were trained with two cues that were both 10 s in duration: One cue was reinforced on every trial (high reinforcement rate, 10 s 100%), but the other cue was reinforced on one in every four trials (low reinforcement rate, 10 s 25%). The low-reinforcement-rate cue was presented four times as often as the high-reinforcement-rate cue; both cues were paired equally often with reinforcement (see Table 1). In wild-type mice, difference scores were greater for the high-reinforcement-rate cue than for the low-reinforcement-rate cue (see Fig. 3a). Consistent with GluA1 having a role in rate calculation beyond any effect on short-term habituation, results revealed that *Gria1*^–/–^ mice had similar difference scores for the high- and low-reinforcement-rate cues (see Fig. 3b). There was a significant Cue × Genotype interaction, *F*(1, 20) = 9.67, *p* = .006, η_*p*_^2^ = .33, 90% CI = [.07, .52]. All other main effects and interactions were nonsignificant (*F*s < 1, *p*s > .70). Further analysis of the significant Cue × Genotype interaction showed that there was a significant effect of cue for wild-type mice, *F*(1, 20) = 37.0, *p* < .001, but the effect of cue failed to reach significance for the *Gria1*^−/−^ mice, *F*(1, 20) = 4.09, *p* = .057. Additionally, difference scores were larger for wild-type mice than for *Gria1*^−/−^ mice for the 10-s 100% cue, *F*(1, 20) = 5.28, *p* = .033, but this was not the case for the 10-s 25% cue, *F*(1, 20) = 0.78, *p* = .39.

**Fig. 4.**
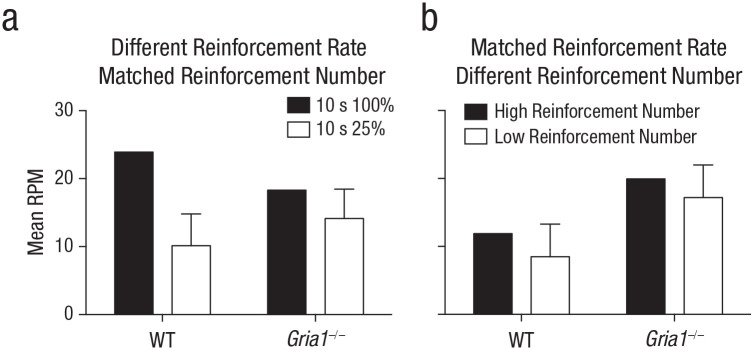
Rates of responding under matched test conditions in Experiments 5 and 6 (a and b, respectively). Each graph shows the mean rate of responding to reinforced cues (number of responses per minute [RPM]) in the first trial of a session, separately for wild-type (WT) and *Gria1*^–/–^ mice. Response rates are collapsed across sessions. In Experiment 5, cues were reinforced either on 100% of trials or on 25% of trials. Error bars indicate 95% confidence intervals for the mean difference between the two cues.

To test whether cue had an effect on learning rather than performance of conditioned responding, we analyzed responding during the first trial of each trial type in each session (see Fig. 4a). There was a significant Cue × Genotype interaction, *F*(1, 20) = 6.05, *p* = .023, η_*p*_^2^ = .23, 90% CI = [.02, .44]. Simple main-effects analysis of the significant interaction revealed that the effect of cue was significantly greater for wild-type mice, *F*(1, 20) = 31.50, *p* < .001, than for *Gria1*^–/–^ mice, *F*(1, 20) = 6.11, *p* = .02.

Although GluA1 deletion impaired sensitivity to reinforcement rate across Experiments 1 to 5, it did not impair sensitivity to the number of times a cue was paired with reinforcement. Indeed, *Gria1*^–/–^ mice were sensitive to reinforcement number under conditions in which wild-type mice were not (Experiment 2; see Figs. 1c and 1d). In Experiment 6, to directly test sensitivity to number of reinforcements, we tested naive mice on a procedure in which cue duration and reinforcement rate were matched but cues differed in number of pairings with reinforcement (see Table 1). Two 10-s cues were both reinforced one in every four trials (reinforcement rate = once every 40 s), but one cue (high reinforcement number) was presented four times more often than the other cue (low reinforcement number). Overall, independent of genotype, difference scores were greater for the high-reinforcement-number cue than for the low-reinforcement-number cue (see Figs. 3c and 3d). There was a significant effect of cue, *F*(1, 20) = 5.57, *p* = .029, η_*p*_^2^ = .22, 90% CI = [.01, .43], and block, *F*(3.33, 66.58) = 9.90, *p* < .001. The effect of genotype failed to reach significance, *F*(1, 20) = 3.60, *p* = .07. All interactions were nonsignificant (*F*s < 1.6, *p*s > .19), demonstrating that GluA1 deletion spares sensitivity to reinforcement number.

To test whether cue had an effect on learning rather than performance of conditioned responding, we analyzed responding during the first trial of each trial type in each session (see Fig. 4b). Mice responded more to the high-reinforcement-number cue than the low-reinforcement-number cue, but the difference was not significant, *F*(1, 20) = 3.61, *p* = .07. *Gria1*^–/–^ mice responded significantly more than wild-type mice, *F*(1, 20) = 6.68, *p* = .02. There was no significant interaction of factors (*F* < 1, *p* = .84). There was no significant effect of genotype for either the high-reinforcement-rate or low-reinforcement-rate cues, largest *F*(1, 20) = 2.51, *p* = .13.

## Discussion

Some models of learning have focused on the temporal dynamics of stimulus processing, such as short-term habituation and memory decay, in order to explain temporal sensitivity in learning (e.g., Brandon, Vogel, & Wagner, 2003; Staddon, 2005). Here, we used a novel test of a time-dependent-processing account: genetic manipulation of short-term habituation. Although GluA1 deletion did affect learning, it was clear that the role of GluA1 was not in determining time-dependent changes in stimulus processing but was instead required for calculation of statistical information about the environment. These results are consistent with theories that propose that learning reflects the encoding of the details and structure of events through symbolic knowledge.

Responding in wild-type mice was sensitive to reinforcement rate under conditions in which that rate was manipulated by the duration of cues or by the probability of reinforcement per trial (see Figs. 1a and 3a, Experiments 1 and 5). When the rate of reinforcement was matched, other factors such as cue duration, probability of reinforcement per trial, and number of pairings with reinforcement failed to affect responding (see Figs. 1c and 1e, Experiments 2 and 3). The sensitivity to reinforcement rate reflected an effect on learning rather than performance. Thus, when the test conditions were matched by comparing responding on the first trial of each trial type per session, wild-type mice responded more to cues that had a high reinforcement rate than to those that had a low reinforcement rate (see Figs. 2a, 2c, and 4a). These results are consistent with other studies in rodents (Holland, 2000), and they support the claim that reinforcement rate is a primary determinant of conditioned responding (Gallistel & Gibbon, 2000; Harris & Carpenter, 2011; Harris et al., 2015).

GluA1 deletion impaired sensitivity to reinforcement rate regardless of whether reinforcement rate was manipulated by cue duration (see Fig. 1b, Experiment 1) or probability of reinforcement per trial (see Fig. 3b, Experiment 5). The fact that GluA1 deletion impaired sensitivity to reinforcement rate when cues were matched for duration (see Fig. 3b, Experiment 5) suggests that GluA1 deletion did not impair rate sensitivity by simply reducing short-term habituation that may have occurred during long-duration cues. Despite impaired rate sensitivity, *Gria1*^–/–^ mice did learn and were sensitive to the number of times a cue was paired with reinforcement. This was the case when cues differed in duration (see Figs. 1d and 1h, Experiments 2 and 4) or frequency of presentation (see Fig. 3d, Experiment 6). Furthermore, *Gria1*^–/–^ mice were sensitive to differences in the number of reinforcements that cues received under conditions in which wild-type mice were not (see Fig. 1d, Experiment 2). Importantly, reinforcement number had an effect on learning rather than on performance of responding (see Figs. 2b and 2d).

The effects observed in Experiments 1 to 6 were found with difference scores in which responding to a nonreinforced, control cue was subtracted from responding to a reinforced cue; scores greater than zero indicate response rates that occurred as a specific consequence of the cue being paired with reinforcement. Importantly, the effects observed with difference scores were also observed with the raw rates of responding to the reinforced cues and, therefore, were not simply due to an effect on response rates to the nonreinforced cues (see Figs. S5 and S6).

It was found that *Gria1*^–/–^ mice responded more than wild-type mice to nonreinforced cues in Experiments 1 to 6. It is unlikely that this baseline difference was the cause of the pattern of results that was observed. Although *Gria1*^–/–^ mice failed to show an effect of cue when cues differed in reinforcement rate but not number of reinforcements (Experiments 1 and 5), they did show an effect of cue when cues differed in the number of reinforcements (Experiments 2, 4, and 6). Furthermore, in Experiment 2, they showed an effect of cue when cues differed in the number of reinforcements under conditions in which wild-type mice did not. Therefore, although there was a baseline difference in responding to nonreinforced cues, the presence or absence of an impairment in *Gria1*^–/–^ mice cannot be explained by this baseline difference.

Although *Gria1*^–/–^ mice were sensitive to the number of pairings with reinforcement, it was clear that learning in *Gria1*^–/–^ mice did not simply increase with every presentation of reinforcement. Responding plateaued at an asymptotic level rather than increasing monotonically over training (see Figs. 1 and 3, Experiments 1–6). The role of the number of pairings of reinforcement across Experiments 1 to 6 (see Figs. 1 and 3) was primarily in determining the rate of acquisition rather than the asymptotic level of performance reached regardless of genotype (see Figs. S8–S11 in the Supplemental Material). Thus, continuously reinforced cues took fewer trials to reach an acquisition criterion than partially reinforced cues. These results are generally consistent with both associative-learning theories, which propose that learning increases with presentations of reinforcement up to a maximum level (e.g., Rescorla & Wagner, 1972), and decision-making theories, which propose that the number of reinforcements determines the rate at which evidence accumulates (e.g., Gallistel & Gibbon, 2000). However, *Gria1*^–/–^ mice did show greater asymptotic levels of responding to a 10-s cue than a 40-s cue when the 10-s cue had been reinforced four times more than the 40-s cue (see Fig. 1h, Experiment 4). In contrast to Experiment 4, in the other experiments in which the number of reinforcements differed between cues (see Figs. 1d and 3d, Experiments 2 and 6), reinforcement number in *Gria1*^–/–^ mice had an effect on the rate of acquisition rather than asymptotic performance (see Figs. S8–S11). A number of variables differed between Experiment 4 (see Fig. 1h), in which asymptotic differences were observed in *Gria1*^–/–^ mice, and the experiments in which there were no differences in asymptotic response rates (see Figs. 1d and 3d, Experiments 2 and 6), such as the overall frequency of reinforcement, the total number of reinforcements per block, and the average interval between trials of the same cue type. Therefore, the cause of this difference was not clear. Nonetheless, across experiments, *Gria1*^–/–^ mice were sensitive to the number of reinforcements (see Figs. 1d, 1h, and 3d, Experiments 2, 4, and 6) but insensitive to reinforcement rate (see Figs. 1b and 3b, Experiments 1 and 5), regardless of rate of acquisition or asymptotic levels of responding (see Figs. S8–S11).

*Gria1*^–/–^ mice failed to weight numeric information by temporal information in order for responding to be sensitive to reinforcement rate. According to an associative analysis, impaired rate sensitivity but preserved sensitivity to reinforcement number may be the consequence of failing to reduce associative strength during periods of nonreinforced cue exposure (see Figs. S16–S18 in the Supplemental Material). This prediction was tested in a supplementary experiment (Experiment 7; see Fig. S1 in the Supplemental Material), but there was no significant evidence for this account, and *Gria1*^–/–^ mice showed normal sensitivity to nonreinforcement.

Symbolic accounts that assume that the statistical properties of events are explicitly encoded provide a different explanation of the effect of GluA1 deletion. Impaired rate sensitivity but preserved sensitivity to the number of reinforcements must be the consequence of a failure to successfully encode temporal durations. This prediction was tested in a supplementary experiment (Experiment 8; see Figs. S2–S4 in the Supplemental Material). *Gria1*^–/–^ mice showed impaired temporal precision, suggesting that impaired sensitivity to rate information may be due to an inability to discriminate between cues of different durations.

The pattern of results was observed using cues that were either 10 s or 40 s in duration and that were reinforced with a probability of either 100% or 25% with either an equivalent or a fourfold difference in reinforcement number. These parameters were chosen because they have been shown to produce a cue-duration effect in normal mice (Austen & Sanderson, 2019). GluA1 deletion impaired the cue-duration effect observed in normal mice using these parameters. Although our analysis assumes that the effect of GluA1 deletion is not dependent on the choice of parameters, it remains to be seen whether a similar pattern of effects is found with other cue durations and other reinforcement rates of the cues relative to the background reinforcement rate.

The collective results fail to support associative accounts of the role of reinforcement rate in learning that appeal to either time-dependent factors in stimulus processing that affect associative strength (Wagner, 1981) or the relative trade-off between increments and decrements in associative strength that occur over cumulative exposure to a cue (Sutton & Barto, 1981; Wagner, 1981). Instead, the results are consistent with decision-making accounts that assume that sensitivity to time, number, and rate information reflects symbolic encoding of these variables and their use in calculating biologically relevant statistical information (Gallistel & Balsam, 2014; Gallistel & Gibbon, 2000; Gibbon, Church, & Meck, 1984).

Although traditional trial-based associative-learning theories have been highly successful in describing the circumstances that lead to learning (Mackintosh, 1975; Pearce & Hall, 1980; Rescorla & Wagner, 1972; Wagner, 1981), their failure to readily describe temporal properties of conditioned responding has led to the proposal that associative learning is not a plausible mechanism for learning at both the psychological and neuronal levels (Gallistel & Balsam, 2014; Gallistel & Gibbon, 2000). Our results provide further support for this claim. However, it is important to note that the dissociation that we demonstrated between associative- and symbolic-encoding accounts of learning was achieved by deletion of the GluA1 AMPA receptor subunit, which is necessary for associative, Hebbian synaptic processes (Malinow & Malenka, 2002; Zamanillo et al., 1999). Therefore, this may contradict the proposal that symbolic encoding cannot be derived from Hebbian processes. It is possible that both associative processes and symbolic representations operate in parallel, dependent on different neural circuits, and there is an interaction between these mechanisms (Petter, Gershman, & Meck, 2018). Alternatively, it has been argued that purely associative mechanisms at the deep learning level may lead to symbolic representations (Mondragon, Alonso, & Kokkola, 2017). Regardless of the merits of these claims, our results demonstrate that simple associative processes are not a sufficient account of learning, and the challenge is now to determine the precise mechanisms by which symbolic encoding is achieved.

## Supplemental Material

sj-pdf-1-pss-10.1177_0956797620960392 – Supplemental material for Dissociating Representations of Time and Number in Reinforcement-Rate Learning by Deletion of the GluA1 AMPA Receptor Subunit in Mice Supplemental material, sj-pdf-1-pss-10.1177_0956797620960392 for Dissociating Representations of Time and Number in Reinforcement-Rate Learning by Deletion of the GluA1 AMPA Receptor Subunit in Mice by Joseph M. Austen, Corran Pickering, Rolf Sprengel and David J. Sanderson in Psychological Science

## Transparency

*Action Editor:* Marc J. Buehner

*Editor:* D. Stephen Lindsay

*Author Contributions*

D. J. Sanderson devised the rationale for the study. D. J. Sanderson and J. M. Austen designed the experiments. J. M. Austen and C. Pickering ran the experiments, and J. M. Austen analyzed the data. All the authors contributed to writing the manuscript and approved the final manuscript for submission.

**Open Practices:** Raw data for this study have been made publicly available via Figshare and can be accessed at https://doi.org/10.6084/m9.figshare.11409447.v1. The design and analysis plans for the study were not preregistered. This article has received the badge for Open Data. More information about the Open Practices badges can be found at http://www.psychologicalscience.org/publications/badges.
