# Supplementary material for: Dissociating Representations of Time and Number in Reinforcement-Rate Learning by Deletion of the GluA1 AMPA Receptor Subunit in Mice
Source: Psychol Sci. 2021 Jan 4;32(2):204–17. doi: 10.1177/0956797620960392 (PMC7883001; doi:10.1177/0956797620960392)
Supplement: sj-pdf-1-pss-10.1177_0956797620960392 – Supplemental material for Dissociating Representations of Time and Number in Reinforcement-Rate Learning by Deletion of the GluA1 AMPA Receptor Subunit in Mice [file sj-pdf-1-pss-10.1177_0956797620960392.pdf]

Supplementary Material for

Dissociating Representations of Time and Number in Reinforcement-Rate Learning by Deletion of the GluA1 AMPA Receptor Subunit in Mice

Joseph M. Austen, Corran Pickering, Rolf Sprengel, & David J. Sanderson

David J. Sanderson / Joseph M Austen
Email: david.sanderson@durham.ac.uk / j.m.austen@durham.ac.uk

This PDF file includes:

Supplementary text
Figures S1 to S18
Tables S1 to S2
Supplementary References

Contents

Supplementary Experiments....4
Experiment 7....4
Method....4
Results....4
Figure S1....5
Table S1....6
Experiment 8....6
Methods....6
Results....7
Figure S2....7
Linear Slopes....7
Gaussian Curve-Fitting....7
Figure S3....8
Within-Trial Start/Stop Analysis....8
Figure S4....9
Table S2....10
Discussion of Experiments 7 and 8....10
Supplementary Statistical Analyses for Experiments 1-6....12
Analysis of Responding to Reinforced and Nonreinforced cues....12
Experiment 1: Different reinforcement rate – matched reinforcement number....12
Figure S5....13
Experiment 2: Matched reinforcement rate – different reinforcement number....14
Experiment 3: Matched reinforcement rate – matched reinforcement number....14
Experiment 4: Different reinforcement rate – different reinforcement number....15
Experiment 5: Different reinforcement rate – matched reinforcement number (10 s only)....15
Figure S6....16
Experiment 6: Matched reinforcement rate – different reinforcement number (10 s only)....16
Analysis of the temporal distribution of responding for the 40 s cue in Experiment 1....17
Figure S7....18
Analysis of Trials to Acquisition and Asymptotic Performance....18
Data Analysis....18
Experiment 1: Different Reinforcement Rate - Matched Reinforcement Number....19
Figure S8....19
Figure S9....19
Experiment 2: Matched Reinforcement Rate - Different Reinforcement Number....20
Experiment 3: Matched Reinforcement Rate - Matched Reinforcement Number....20

Experiment 4: Different Reinforcement Rate - Different Reinforcement Number 20
Experiment 5: Different Reinforcement Rate - Matched Reinforcement Number 20
Figure S10 21
Figure S11 21
Experiment 6: Matched Reinforcement Rate - Different Reinforcement Number 21
Timing Analysis for Experiments 1-6 21
Figure S12 22
Figure S13 23
Experiment 1: Different Reinforcement Rate – Matched Reinforcement Number 23
Figure S14 23
Experiment 2: Matched Reinforcement Rate – Different Reinforcement Number 24
Experiment 3: Matched Reinforcement Rate – Matched Reinforcement Number 24
Experiment 4: Different Reinforcement Rate – Different Reinforcement Number 24
Experiment 5: Different Reinforcement Rate – Matched Reinforcement Number (10 s only) 24
Figure S15 24
Experiment 6: Matched Reinforcement Rate – Different Reinforcement Number (10 s only) 24
Associative Learning Simulations 25
Model 25
Cue Duration Experiments – Experiment 1-4 25
Figure S16 26
Matched Cue Duration Experiments – Experiment 5-6 26
Figure S17 27
Extinction – Experiment 7 27
Figure S18 28
Supplementary References 29

Supplementary Experiments

Experiment 7

Experiments 1-6 demonstrate that learning in normal mice is sensitive to the rate of reinforcement, regardless of whether reinforcement rate is manipulated by the duration of the cue or the probability of reinforcement per trial. Deletion of GluA1 qualitatively changed learning, as indicated from the difference scores and the raw rates of responding, from being dependent on reinforcement rate to being dependent on the number of pairings with reinforcement, revealing that GluA1 is necessary for weighting numeric information by temporal information for the calculation of reinforcement rate. Sensitivity to temporal information in the predictive learning procedures reflected extraction of statistical information from events. While this may reflect explicit encoding of numeric and temporal variables (Balsam & Gallistel, 2009; Gallistel & Gibbon, 2000), it may also be achieved by the balance of increments and decrements in associative strength across cumulative exposure to cues (Schmajuk, Gray, & Lam, 1996; Sutton & Barto, 1981, 1990). Thus, an error correction rule implemented in an iterative manner over time achieves rate-sensitivity by reducing associative strength during periods of nonreinforced cue exposure (see Figures S16-17). This associative model predicts that GluA1 deletion impairs the decrements in learning during nonreinforcement (i.e., learning through negative prediction error (Rescorla & Wagner, 1972)). We tested this prediction in Experiment 7 by training mice on an extinction procedure in which a previously trained cue was presented in the absence of reinforcement. Failure to extinguish conditioned responding in *Gria1*$^{-/-}$ mice would support the associative hypothesis (see simulation of extinction in Figure S18).

Method

Procedure

Twelve *Gria1*$^{-/-}$ (6 female, 6 male) and 12 wild-type (6 female, 6 male) mice (free-feeding weights: 17.4 – 33.4 g) initially received 10 sessions of training, one session per day, in which three 10 s cues were presented eight times each per session. Trials were separated by a fixed interval of 120 s (cue offset to cue onset). Two of the cues were reinforced (by presentation of a sucrose pellet at the termination of the cue) on every trial and one cue was nonreinforced. The order of trials was random with the constraint that there was an equal number of each trial type every six trials. In the second stage, which lasted for eight sessions, all procedures were the same except that one of the previously reinforced cues was now nonreinforced (extinction). The other previously reinforced cue continued to be reinforced (non-extinction). The three stimuli were white noise, tone, and clicker. The allocation of stimuli to trial types was counterbalanced within genotype and sex.

Results

Similar to Experiments 1-6, in order to have a measure of responding that indicated performance above baseline, the analysis below reports response rates that were converted to difference scores in which the response rate for nonreinforced cues was subtracted from the response rates for the reinforced cues. *Gria1*$^{-/-}$ mice responded at a significantly higher rate for the nonreinforced cue than wild-type mice in both the first stage of acquisition and in the extinction phase (p-values $\leq$ 0.012, see Table S1).

Difference Scores: Wild-type and Gria1$^{-/-}$ mice showed similar acquisition in terms of difference scores during the first stage (Figure S1). A repeated measures ANOVA of cue (non-extinction or extinction) x genotype (*Gria1*$^{-/-}$ or wild-type) x block on the difference scores (responding to the CS+ minus responding to the CS−) showed a significant main effect of block, $F(3.16,69.60) = 24.1$, $p < 0.001$. All other main effects and interactions were non-significant, F-values < 2.2, p-values > 0.06.

During the extinction phase both wild-type and *Gria1*$^{-/-}$ mice showed extinction of conditioned responding to the non-reinforced cue in terms of reductions in differences scores (Figure S1). A repeated measures ANOVA of cue (non-extinction or extinction) x genotype (*Gria1*$^{-/-}$ or wild-type) x block on the difference scores (responding to the CS+ minus responding to the CS−) showed significant main effects of cue, $F(1,22) = 38.6$, $p < 0.001$, and block, $F(3.67,80.76) = 2.64$, $p = 0.044$, and a significant cue x block interaction, $F(3.71,81.72) = 11.7$, $p < 0.001$. All other main effects and interactions were non-significant, F-values < 2.6, p-values > 0.12. Further analysis of the significant cue x block interaction showed a significant effect of cue on blocks 2-8 of the extinction phase, F-values > 15.6, p-values < 0.001. In order to assess whether the results provided evidence for a lack of difference in terms of the effect of extinction between genotypes we calculated Bayes factors using Bayesian ANOVA for the difference scores for the extinguished cue across blocks. For the comparison of the effect of genotypes $BF_{10} = 0.290$ and for the interaction between genotype and block $BF_{Incl} = 0.109$. Therefore, the lack of impairment in *Gria1*$^{-/-}$ mice fails to support the associative account of the role of GluA1 in reinforcement rate learning.

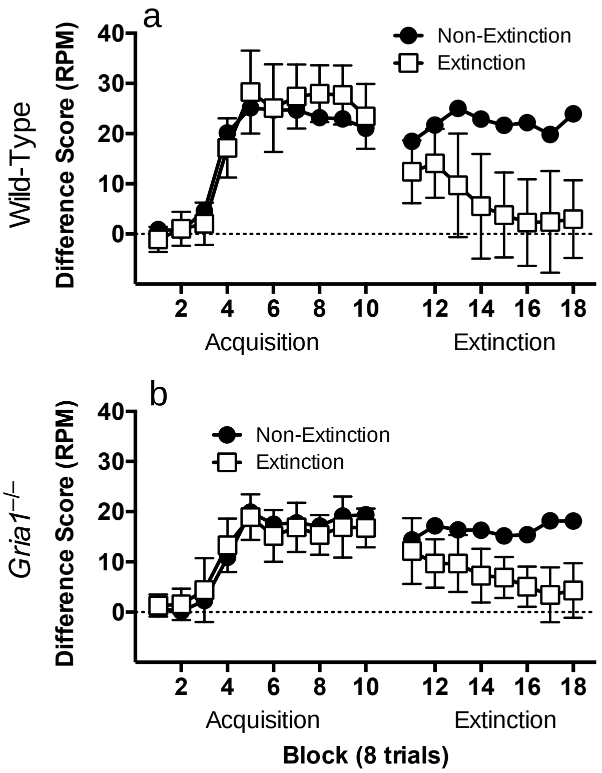

Figure S1. GluA1 deletion does not impair extinction of conditioned responding (Experiment 7). Mean responding (food magazine entries) is shown minus the mean response rate to a nonreinforced cue (difference score, responses per minute, RPM). Panels a and b: during the acquisition phase (blocks 1-10) mice received training with two 10 s cues that were reinforced on every trial. During the extinction phase (blocks 11-18) the extinction cue was no longer reinforced, but the non-extinction cue continued to be reinforced. Error bars on the white squares indicate the 95% confidence interval for the mean difference between the two cues.

Non-reinforced cue: During acquisition Gria1$^{-/-}$ mice responded to the non-reinforced cue more than wild-type mice throughout the acquisition stage (see Table S1 for means and SEMs). A repeated measures ANOVA of genotype (*Gria1*$^{-/-}$ or wild-type) x block on responding to the non-reinforced cue showed significant main effects of genotype, $F(1,22) = 36.4$, $p < 0.001$, and block, $F(4.27,93.82) = 10.6$, $p < 0.001$, but no significant genotype x block interaction, $F(4.27,93.82) = 1.84$, $p = 0.12$.

Gria1$^{-/-}$ mice continued to respond to the non-reinforced cue more than wild-type mice throughout the extinction stage (see Table S1 for means and SEMs). A repeated measures ANOVA of genotype (*Gria1*$^{-/-}$ or wild-type) x block on responding to the non-reinforced cue showed significant main effects of genotype, $F(1,22) = 7.54$, $p = 0.012$, and block, $F(2.89,63.58) = 5.53$, $p = 0.002$, but no significant genotype x block interaction, $F(2.89,63.58) = 1.27$, $p = 0.29$.

Table S1. *Mean (SEM) responses per minute (RPM) during the CS− periods of the acquisition and extinction stages of the extinction experiment (Experiment 7).*

**Stage**	**Genotype**	**Block**									
		**1**	**2**	**3**	**4**	**5**	**6**	**7**	**8**	**9**	**10**
Acquisition	*Gria1*$^{-/-}$	11.5 (0.9)	24.4 (3.1)	23.5 (2.5)	18.5 (2.1)	15.9 (1.9)	14.3 (3.3)	11.3 (2.3)	12.5 (2.0)	10.5 (1.3)	8.4 (1.8)
	Wild-type	5.4 (0.8)	9.4 (1.6)	12.5 (1.6)	11.7 (2.0)	8.3 (1.3)	8.6 (1.3)	6.3 (1.4)	5.0 (1.0)	5.8 (1.5)	4.6 (1.1)
Extinction	*Gria1*$^{-/-}$	8.9 (2.5)	7.0 (2.0)	6.3 (1.9)	6.1 (1.6)	3.6 (0.9)	4.4 (0.9)	4.2 (1.1)	3.0 (0.7)		
	Wild-type	4.3 (1.0)	1.4 (0.4)	1.8 (0.4)	2.8 (0.8)	1.9 (0.5)	2.6 (0.9)	1.3 (0.5)	0.4 (0.4)		

Experiment 8

Experiment 7 failed to show that GluA1 deletion impaired extinction learning suggesting that the impaired sensitivity to reinforcement rate in *Gria1*$^{-/-}$ mice is not due to impaired sensitivity to nonreinforcement. An alternative explanation is that GluA1 is necessary for timing of cue duration and that this leads to an impaired ability to calculate reinforcement rate. In Experiment 8, in order test the role of GluA1 in temporal learning, we trained naïve mice on a peak procedure in which a 30 s cue was reinforced 10 s after the onset of the cue on 75% of trials. The remaining 25% of trials served as probe trials that allowed for the assessment of the timing of responding across the duration of the cue.

Methods

Procedure

Fourteen *Gria1*$^{-/-}$ (7 female, 7 male) and 23 wild-type (12 female, 11 male) mice (free-feeding weights: 20.9 – 32.6 g) received 12 sessions of training, one session per day, in which two 30 s cues (white noise and clicker) were presented twelve times each per session. Trials were separated by a fixed interval of 120 s (cue offset to cue onset). One of the cues was reinforced (by presentation of a sucrose pellet 10 s after cue onset) on 75% of trials and the other cue was nonreinforced. The order of trials was random with the constraint that there was an equal number of each trial type every eight trials, with the reinforced cue being reinforced on three of its four trials. The allocation of stimuli to trials types was approximately counterbalanced within genotype and sex.

Data Analysis

Timing of responding was assessed in two ways: linear slopes and Gaussian curve-fitting. Linear slopes: Responding during the first 10 s of the reinforced cue (i.e., prior to the delivery of reinforcement) was divided into ten equal time periods (i.e., ten one-second time-bins) and averaged across all sessions of the experiment. These data were then normalised to show the proportion of responding an animal made during each of the ten time-bins. The linear gradients of these normalised data were then calculated to provide an indication of the extent that responding to cues was being timed (i.e., the steeper the gradient the more the animals timed their responding to the delivery of the US).

Gaussian curve-fitting: Following the procedure used by Tam, Jennings, and Bonardi (2015) responding during each 1 s time-bin of the entire 30 s of the non-rewarded presentations of the reinforced cue was averaged across all trials and sessions and then smoothed over four 1 s bins using a running average (i.e., the mean of 1-4 s, 2-5 s etc.) in order to reduce the influence of second by second chance fluctuations in response rates . A Gaussian model (Eq. 1) was then fitted to the timing distribution of each animal. Here, R_i is the conditioned responding in smoothed time-bin i, A is the peak rate of responding, B is the spread of the distribution, C is the time-bin at which the peak rate occurs (the central tendency, peak time), and x_i is the time since CS onset in smoothed time-bin i.

$$R_i = Ae^{-\frac{1}{2}\left(\frac{x_i - C}{B}\right)^2} \qquad \text{Eq. 1}$$

The different measures of timing were analysed using Mann Whitney U tests.

Results

The mean rate of responding per second across all probe trials is shown in Figure S2a.

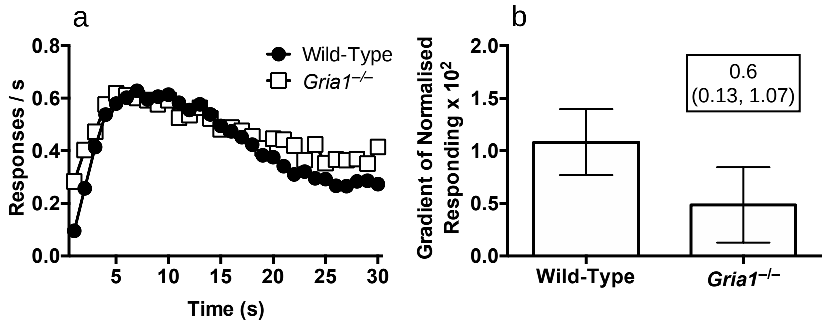

Figure S2. GluA1 deletion impairs timing of conditioned responses. Mice were trained with a 30 s cue that was reinforced 10 s after the onset of the cue. On probe trials the cue was non-reinforced in order to measure the distribution of responding around the time of the peak in responding (Experiment 8). Panel a: response rates per second as a function of time within nonreinforced probe trials. Panel b: GluA1 deletion reduced the gradient of the linear slopes fitted to the normalized response rates to the first 10 s of the 30 s cue (prior to reward delivery). Error bars indicate the 95% confidence interval. The inset text reports the mean difference between genotypes and the lower and upper extent of the 95% confidence interval.

Linear Slopes

Wild-type mice showed steeper linear gradients of normalised responding than *Gria1*$^{-/-}$ mice, $F(1,35) = 6.54$, $p = 0.015$ (see Figure S2b).

Gaussian Curve-Fitting

A Gaussian model with three parameters was fitted to the conditioned responding. Genotype comparisons for each of these parameters (peak rate, spread, and central tendency (i.e., peak time)) were conducted using Mann-Whitney U tests. There was no effect of genotype on peak rate, $U = 125$, $p = 0.27$ (Figure S3a), or on central tendency (peak time), $U = 162$, $p = 1.0$ (Figure S3b), but there was a significant effect of genotype on spread, $U = 62.0$, $p = 0.001$, with *Gria1*$^{-/-}$ mice producing timing distributions with larger spreads than wild-type mice (Figure S3c). Using the parameters derived from the Gaussian model it was possible to calculate the error in the peak time compared to the time of

reinforcement (i.e., √[peak time minus time of reinforcement]^2), and the coefficient of variation (spread/peak time). There was no significant effect of genotype for either measure: peak time error, U = 148, p = 0.70 (Figure S3d); coefficient of variation (spread/peak time), U = 123.5, p = 0.24 (Figure S3e). R^2, the coefficient of determination of the regression model was calculated (Kvalseth, 1985). The values of R^2 (overall median = 0.71) indicate that the Gaussian model was a good fit for the observed data. Additionally, the R^2 values were significantly higher for wild-type mice, U = 227, p = 0.039 (Figure S3f) consistent with wild-type mice showing superior temporal control of responding.

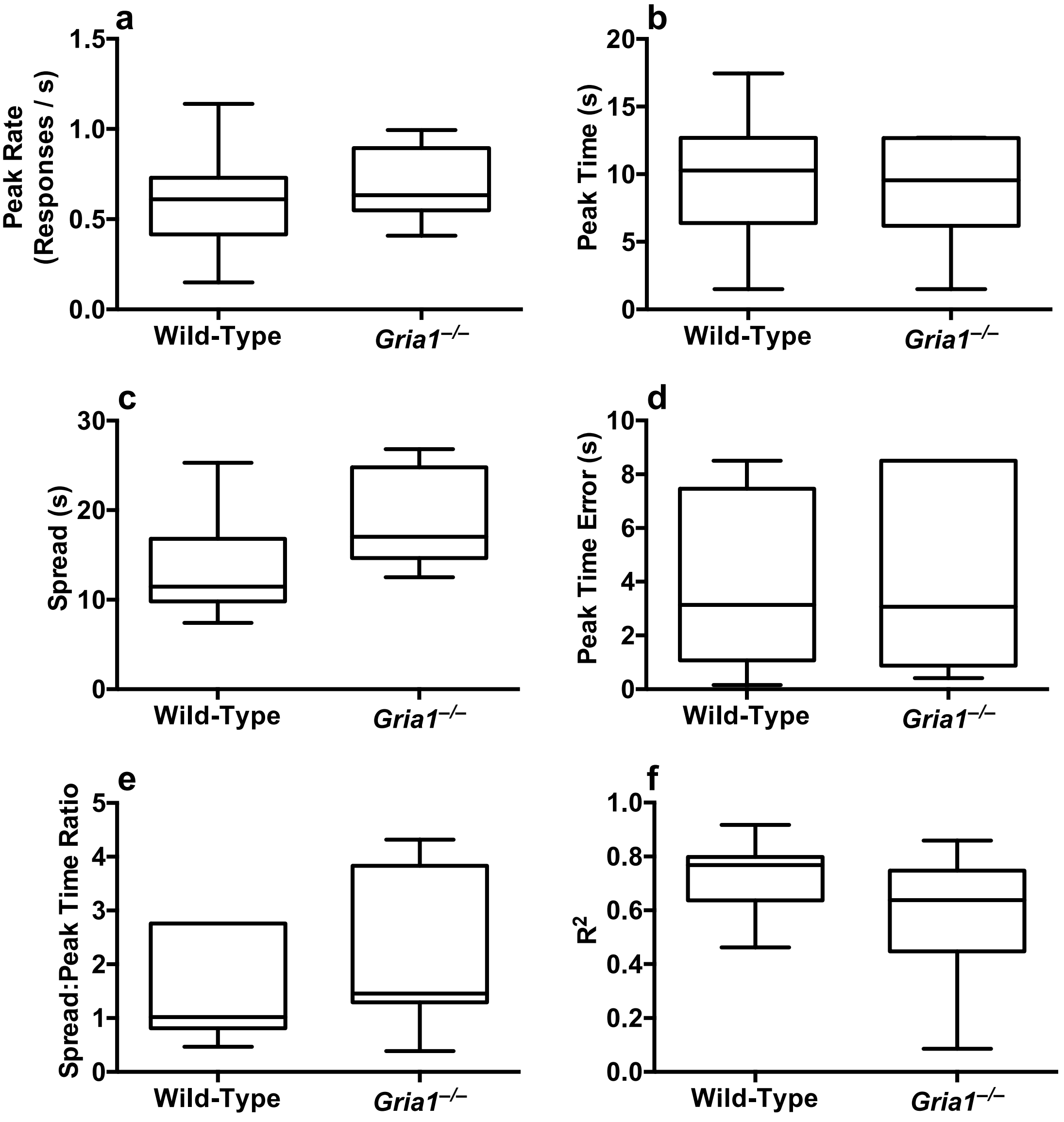

Figure S3. Timing parameters derived from the Gaussian curve-fitting analysis of the peak procedure. a: peak response rate. b: peak time. c: spread. d: peak time error. e: spread as a ratio of peak time. f: coefficient of determination of the Gaussian model, R^2. Boxplots show median and IQR. Whiskers indicate 1.5IQR.

Within-Trial Start/Stop Analysis

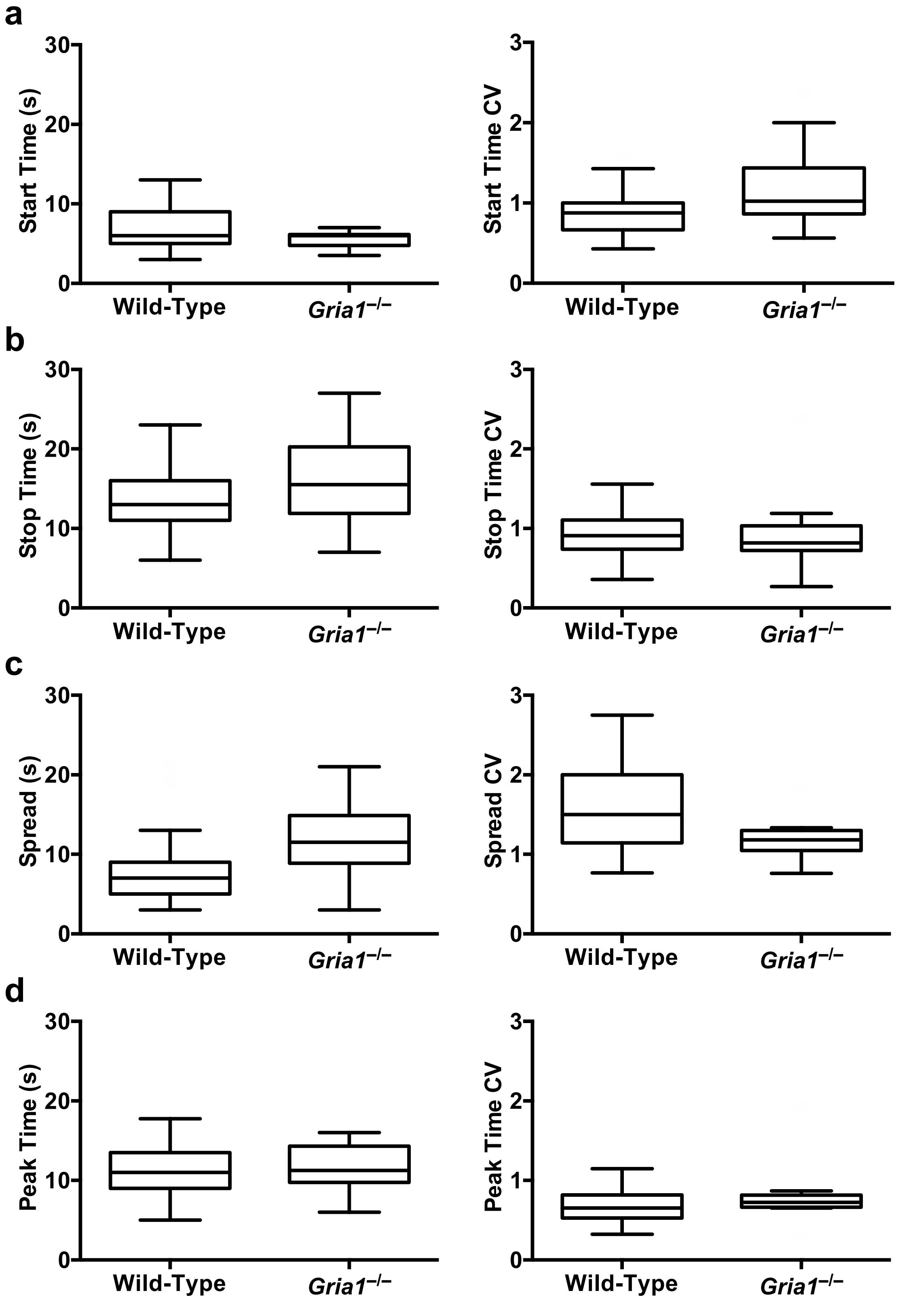

Figure S4. Within-trial analysis of peak procedure probe trials (Experiment 8). a: start-time (left panel) and start-time CV (right panel). b: stop-time (left panel) and stop-time CV (right panel). c: spread (left panel) and spread CV (right panel). d: peak time (left panel) and peak time CV (right panel). Boxplots show median and IQR. Whiskers indicate 1.5IQR.

Responses per second were also analysed for individual probe trials in order to determine when responding started and stopped within a trial. Responses per second were smoothed by taking a running average of response rates for three 1-second time-bins such that the smoothed response rate for the 2nd 1-second time-bin was the mean of the response rates for seconds 1-3, 3rd 1-second time-bin was the mean of response rates for seconds 2-4 etc. The peak response rate achieved within a trial was recorded and the time point at which the peak response was first achieved was deemed the start-time and the time point at which it was last achieved was deemed the stop-time. The median start and stop times across all probe trials were calculated for each mouse. The peak response time for each trial was calculated as the midpoint between the start and stop times. The spread of responding was the difference between the stop-time and the start-time. The coefficient of variance (CV) for each measure was calculated by dividing the median duration by the interquartile range for each mouse.

The results are shown in Figure S4. Although start-times occurred sooner and stop-times later in *Gria1*$^{-/-}$ mice than wild-type mice, they did not significantly differ (see Table S2). There was no significant difference in the peak response time, but the spread of responding was significantly greater in *Gria1*$^{-/-}$ mice than wild-type mice. Analyses of the CV for each measure failed to reveal any significant differences between genotypes in the variability of each measure.

*Table S2***.** Mann-Whitney U values and p-values for the start time, stop time, spread, peak time and coefficient of variance for each measure.

Measure	Mann-Whitney U	N	p-value
Start Time	214	37	0.101
Start Time CV	99	37	0.053
Stop Time	122	37	0.231
Stop Time CV	177	37	0.610
Spread	84	37	0.015
Spread CV	214	37	0.101
Peak Time	152	37	0.793
Peak Time CV	112	37	0.130

Discussion of Experiments 7 and 8

The fact that wild-type mice were sensitive to reinforcement rate and that *Gria1*$^{-/-}$ mice were sensitive to the number of times a cue was paired with reinforcement suggests that GluA1 is necessary for weighting numeric information by temporal information in order for rate-sensitivity to be achieved. Real-time associative accounts of learning in which associations are updated continuously propose that rate sensitivity is a result of increments in associative strength during periods of reinforcement and losses of associative strength or discounting of reinforcement during nonreinforcement (Sutton & Barto, 1990; Wagner, 1981). It follows from this account that intact sensitivity to the number of reinforcements, but not reinforcement rate, must reflect impaired ability to reduce learning during nonreinforcement (see Figure S18). If this is the case, then GluA1 deletion should impair the loss of associative strength during extinction in which a previously reinforced cue is no longer reinforced. This was not the case, however, and *Gria1*$^{-/-}$ mice showed normal levels of extinction across nonreinforced exposure (Figure S1, Experiment 7).

Symbolic accounts of learning propose that quantifiable properties of cues are explicitly encoded. For example, Rate Estimation Theory proposes that animals store memories of temporal and numeric variables in order to calculate rate information (Gallistel & Gibbon, 2000). This account would, therefore, assume that impaired reinforcement rate-sensitivity, but intact sensitivity to the number of reinforcements, must reflect that *Gria1*$^{-/-}$ mice fail to weight the number of reinforcements by the cumulative cue duration. This may be due to a failure to time cues appropriately. The ability of *Gria1*$^{-}$

/– mice to time responding was assessed using the peak procedure. It was found that while *Gria1*$^{-/-}$ mice showed a peak of responding close to the time of reinforcement that was similar to wild-type mice, the distribution of responding around the peak time was broader for *Gria1*$^{-/-}$ mice than wild-type mice (Figure S3c, Experiment 8). Although not significant, *Gria1*$^{-/-}$ mice tended to start responding sooner and cease responding later in a trial than wild-type mice (see Figure S4a-b), but the duration of responding within trials was significantly longer in *Gria1*$^{-/-}$ mice than wild-type mice (see Figure S4c). Importantly, both genotypes showed similar levels of variability in the timepoints of starting and stopping responding across trials (see Figure S4a-b) suggesting that the reduced precision of timing in *Gria1*$^{-/-}$ mice was not due to increased variability of decision thresholds for responding. The timing precision impairment was also evident from analyses of Experiments 1-6 (see Figures S14-15). This timing precision impairment may result in an inability to discriminate between temporal durations and therefore between cues that have different reinforcement rates.

Supplementary Statistical Analyses for Experiments 1-6

Analysis of Responding to Reinforced and Nonreinforced cues

Experiment 1: Different reinforcement rate – matched reinforcement number

Reinforced cues: Wild-type mice showed greater responding to the 10 s cue than the 40 s cue, but this difference was smaller in *Gria1*$^{-/-}$ mice (see Figure S5a). A repeated measures ANOVA of cue duration (10 s or 40 s) x genotype (*Gria1*$^{-/-}$ or wild-type) x counterbalance (10 s cues auditory or visual; nuisance factor) x block was performed. The effect of cue was significant, $F(1,23) = 19.18$, $p < 0.001$, and significantly interacted with genotype, $F(1,23) = 9.07$, $p = 0.006$, and block, $F(4.56,104.98) = 5.46$, $p < 0.001$. There was a significant effect of genotype, $F(1,23) = 15.78$, $p = 0.001$, and genotype by block interaction, $F(3.17,72.98) = 2.45$, $p = 0.067$. There was no significant three-way interaction between cue, block and genotype, $F(4.56,104.98) = 1.63$, $p = 0.16$. Simple main effects analysis of the significant cue by genotype interaction revealed that wild-type mice showed greater responding to the 10 s cue than the 40 s cue, $F(1,23) = 28.45$, $p < 0.001$, but *Gria1*$^{-/-}$ mice did not, $F < 1$, $p = 0.35$. *Gria1*$^{-/-}$ mice showed greater responding than wild-type mice for both the 10 s and 40 s cues, smallest $F(1,23) = 5.94$, $p = 0.02$.

Non-reinforced cues: Gria1$^{-/-}$ and wild-type mice both responded slightly more to the non-reinforced 10 s cue than to the non-reinforced 40 s cue. *Gria1*$^{-/-}$ mice responded to the non-reinforced cues more than wild-type mice (see Figure S5a). A repeated measures ANOVA of cue duration (10 s or 40 s) x genotype (*Gria1*$^{-/-}$ or wild-type) x counterbalance (10 s cues auditory or visual; nuisance factor) x block on responding to the non-reinforced cues showed significant main effects of cue duration, $F(1,23) = 26.4$, $p < 0.001$, block, $F(2.52,57.89) = 8.28$, $p < 0.001$, and genotype, $F(1,23) = 28.4$, $p < 0.001$, and significant interactions between block and genotype, $F(2.52,57.89) = 3.25$, $p = 0.035$, and cue duration and block, $F(5.19,119.36) = 5.34$, $p < 0.001$. The cue duration x genotype interaction approached significance, $F(1,23) = 3.58$, $p = 0.071$, but the cue duration x block x genotype interaction was not significant, $F(5.19,119.36) = 1.38$, $p = 0.24$.

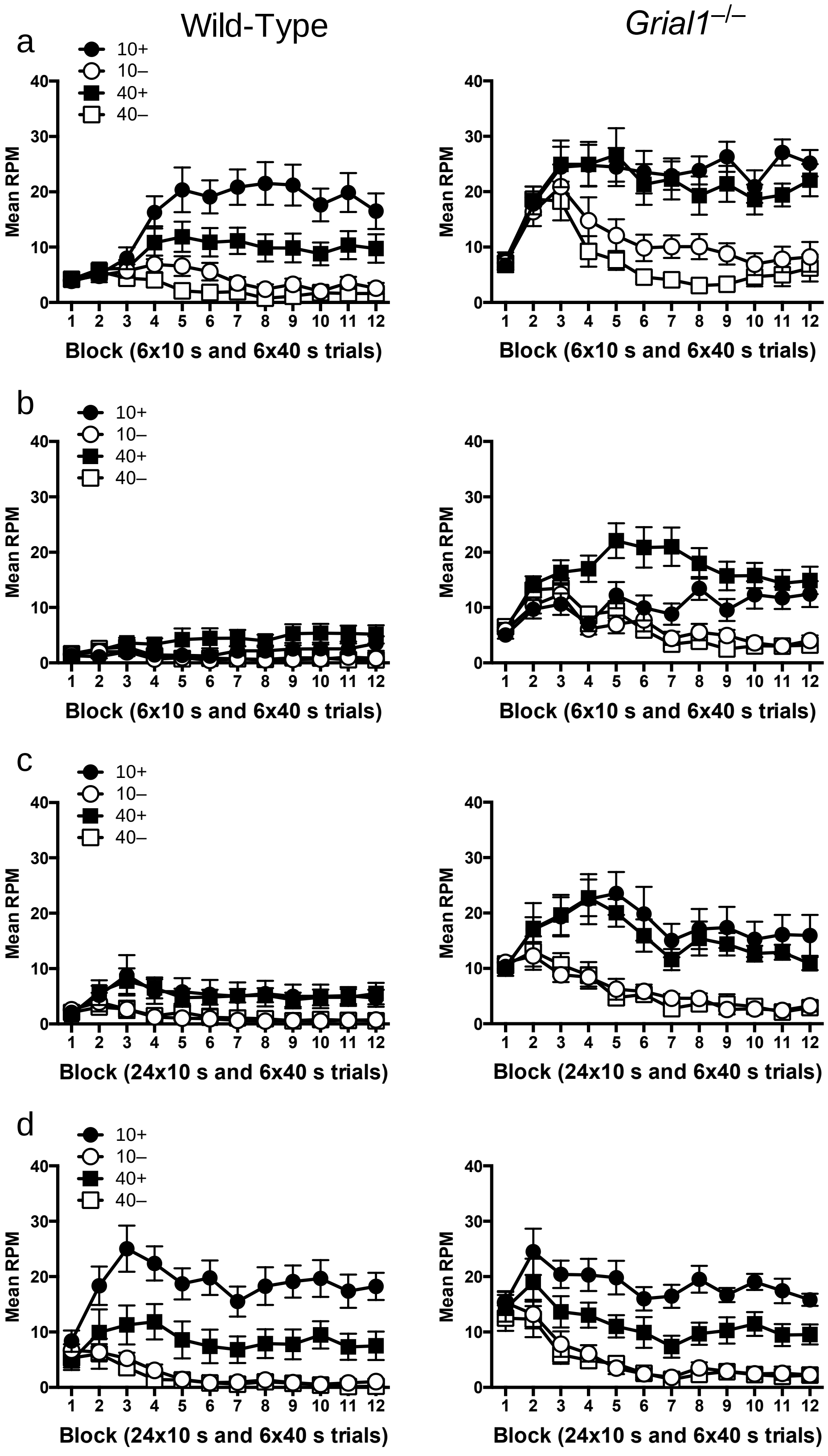

Figure S5. Mean rates of responding per minute (RPM) for reinforced (+) and nonreinforced (–) cues in the cue duration experiments, Experiments 1-4 in panels a-d respectively. Error bars indicated ±SEM.

Experiment 2: Matched reinforcement rate – different reinforcement number

Reinforced cues: *Gria1*$^{-/-}$ mice responded more to the continuously reinforced 40 s cue than the partially reinforced cue, but this difference was less apparent in wild-type mice (see Figure S5b). A repeated measures ANOVA of cue duration (10 s or 40 s) x genotype (*Gria1*$^{-/-}$ or wild-type) x counterbalance (10 s cues auditory or visual; nuisance factor) x block was performed. The effect of cue was significant, $F(1,28) = 17.04$, $p < 0.001$, and significantly interacted with block, $F(4.10,114.90) = 4.38$, $p = 0.002$. The cue by genotype interaction was not significant, $F(1,28) = 4.10$, $p = 0.05$, but there was a significant cue by genotype by block interaction, $F(4.10,114.90) = 2.80$, $p = 0.028$. The nature of the three-way interaction was analysed by conducting separate ANOVAs for each genotype. For wild-type mice, there was no significant cue by block interaction, $F < 1$, $p = 0.56$, and no significant effect of cue, $F(1,14) = 3.45$, $p = 0.08$. For *Gria1*$^{-/-}$ mice, there was a significant cue by block interaction, $F(3.95,55.28) = 4.29$, $p = 0.004$. Simple main effects analysis of the cue by block interaction revealed that *Gria1*$^{-/-}$ mice showed significantly greater responding to the continuously reinforced 40 s cue than the partially reinforced 10 s cue on blocks 2-7, smallest $F(1,14) = 15.23$, $p = 0.002$.

Non-reinforced cues: Gria1$^{-/-}$ mice responded to the non-reinforced cues more than wild-type mice, but there was little effect of cue duration on responding to the nonreinforced cues (see Figure S5b). A repeated measures ANOVA of cue duration (10 s or 40 s) x genotype (*Gria1*$^{-/-}$ or wild-type) x counterbalance (10 s cues auditory or visual; nuisance factor) x block on responding to the non-reinforced cues showed significant main effects of block, $F(3.94,110.36) = 18.4$, $p < 0.001$, and genotype, $F(1,28) = 52.8$, $p < 0.001$, and significant block x genotype, $F(3.94,110.36) = 9.48$, $p < 0.001$, cue duration x block, $F(3.72,104.27) = 3.22$, $p = 0.018$, and cue duration x block x genotype, $F(3.72,104.27) = 2.58$, $p = 0.045$, interactions. All other main effects and interactions were nonsignificant, F-values < 1, p-values > 0.5. Further analysis of the significant three-way interaction between cue duration, block and genotype was achieved by separate ANOVAs for each genotype. For *Gria1*$^{-/-}$ mice, there was a significant main effect of block, $F(3.82,53.47) = 14.6$, $p < 0.001$, and a significant cue duration x block interaction, $F(3.22,45.09) = 3.13$, $p = 0.032$. The main effect of cue duration was not significant, $F(1,14) = 0.049$, $p = 0.83$. Further analysis of the significant cue duration x block interaction showed a significant effect of cue duration on blocks 2, 4, and 9, F-values > 6.2, p-values < 0.03. Wild-type mice showed a significant main effect of block, $F(3.07,42.91) = 6.22$, $p = 0.001$, but no significant main effect of cue duration, $F(1,14) = 2.04$, $p = 0.18$, and no significant cue duration x block interaction, $F(4.81,67.32)$ 1.14, $p = 0.35$.

Experiment 3: Matched reinforcement rate – matched reinforcement number

Reinforced cues: Both genotypes showed little difference in responding to the 10 s and 40 s cues (see Figure S5c). A repeated measures ANOVA of cue duration (10 s or 40 s) x genotype (*Gria1*$^{-/-}$ or wild-type) x counterbalance (10 s cues auditory or visual; nuisance factor) x block was performed. The effect of cue was not significant, $F < 1$, $p = 0.80$. There was a significant effect of block, $F(2.48,32.17) = 4.70$, $p = 0.011$, and genotype, $F(1,13) = 34.09$, $p < 0.001$. There were no significant interactions of factors, p values > 0.16.

Non-reinforced cues: Gria1$^{-/-}$ mice responded to the non-reinforced cues more than wild-type mice, but there was little effect of cue duration on responding to the non-reinforced cues (see Figure S5c). A repeated measures ANOVA of cue duration (10 s or 40 s) x genotype (*Gria1*$^{-/-}$ or wild-type) x counterbalance (10 s cues auditory or visual; nuisance factor) x block on responding to the non-reinforced cues showed significant main effects of block, $F(2.35,30.49) = 17.9$, $p < 0.001$, and genotype, $F(1,13) = 46.5$, $p < 0.001$, and a significant block x genotype interaction, $F(2.35,30.49) = 6.25$, $p = 0.004$. All other main effects and interactions were non-significant, F-values < 1, p-values > 0.4.

Further analysis of the significant block x genotype interaction showed a significant effect of genotype for all blocks, F-values > 9.7, p-values < 0.01.

Experiment 4: Different reinforcement rate – different reinforcement number

Reinforced cues: Both genotypes responded more to the 10 s cue than the 40 s cue (see Figure S5d). A repeated measures ANOVA of cue duration (10 s or 40 s) x genotype (*Gria1*$^{-/-}$ or wild-type) x counterbalance (10 s cues auditory or visual; nuisance factor) x block was performed. The effect of cue was significant, $F(1,14) = 64.65$, $p < 0.001$. There was a significant effect of block, $F(3.46,48.41) = 4.91$, $p = 0.003$. There were no other significant main effects of interactions, p values > 0.14.

Non-reinforced cues: Gria1$^{-/-}$ mice responded to the non-reinforced cues more than wild-type mice, but there was little effect of cue duration on responding to the nonreinforced cues (see Figure S5d). A repeated measures ANOVA of cue duration (10 s or 40 s) x genotype (*Gria1*$^{-/-}$ or wild-type) x counterbalance (10 s cues auditory or visual; nuisance factor) x block on responding to the non-reinforced cues showed significant main effects of block, $F(2.54,35.54) = 28.5$, $p < 0.001$, and genotype, $F(1,14) = 12.2$, $p = 0.004$. All other main effects and interactions were non-significant, F-values < 4.3, p-values > 0.05.

Experiment 5: Different reinforcement rate – matched reinforcement number (10 s only)

Reinforced cues: Both genotypes responded more to the 10 s 100% cue than the 10 s 25% cue, but this difference was greater in wild-type mice than *Gria1*$^{-/-}$ mice (see Figure S6a). A repeated measures ANOVA of cue (1/10 s or 1/40 s) x genotype (*Gria1*$^{-/-}$ or wild-type) x counterbalance (1/10 s cue auditory or visual; nuisance factor) x block was performed. There was a significant effect of cue, $F(1,20) = 58.11$, $p < 0.001$, that significantly interacted with genotype, $F(1,20) = 8.52$, $p = 0.008$. There was a significant effect of block, $F(3.04,60.69) = 10.05$, $p < 0.001$, and cue by block interaction, $F(4.76,95.21) = 10.85$, $p < 0.001$. There were no other significant main effects or interactions of factors, p values > 0.1. Simple main effects analysis of the significant cue by genotype interaction demonstrated that the effect of cue was significantly greater for wild-type mice, $F(1,20) = 51.24$, $p < 0.001$, than for *Gria1*$^{-/-}$ mice, $F(1,20) = 12.08$, $p = 0.002$. The effect of genotype was not significant for either cue, largest $F(1,20) = 2.80$, $p = 0.11$.

Non-reinforced cues: Gria1$^{-/-}$ mice responded to the non-reinforced cues more than wild-type mice early in training and responding to the non-reinforced 10 s 100% cue was higher than to the nonreinforced 10 s 25% cue for both genotypes (see Figure S6a). A repeated measures ANOVA of cue (1/10 s or 1/40 s) x genotype (*Gria1*$^{-/-}$ or wild-type) x counterbalance (1/10 s cue auditory or visual; nuisance factor) x block on responding to the non-reinforced cues showed significant main effects of cue, $F(1,20) = 27.9$, $p < 0.001$, block, $F(3.63,72.62) = 14.9$, $p < 0.001$, and genotype, $F(1,20) = 4.95$, $p = 0.038$, and significant genotype x block, $F(3.63,72.62) = 4.02$, $p = 0.007$, and cue x block, $F(4.54,90.74) = 3.53$, $p = 0.007$, interactions. All other interactions were non-significant, F-values < 1, p-values > 0.6. Further analysis of the significant genotype x block interaction showed a significant effect of genotype for blocks 1 and 2, F-values > 4.9, p-values < 0.04. Further analysis of the significant cue x block interaction showed a significant effect of cue on blocks 2-9 and 11-12, F-values > 6.4, p-values < 0.02.

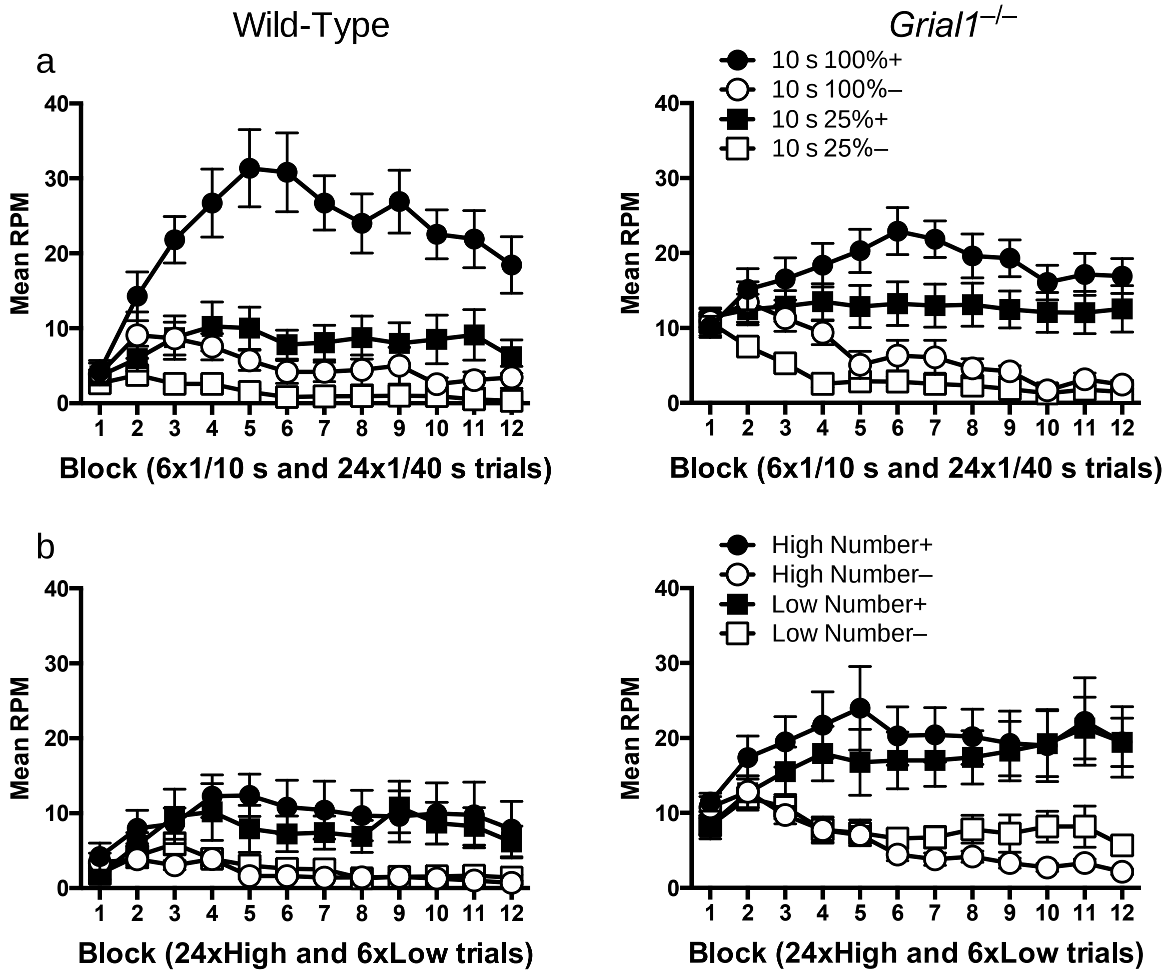

Figure S6. Mean rates of responding per minute (RPM) for reinforced (+) and nonreinforced (–) cues in the matched cue duration experiments, Experiments 5 and 6 in panel a-b respectively. Error bars indicated ±SEM.

Experiment 6: Matched reinforcement rate – different reinforcement number (10 s only)

Reinforced cues: There was little difference in response rates for the high and low reinforcement number cues (see Figure S6b). A repeated measures ANOVA of cue (high reinforcement number or low reinforcement number) x genotype (*Gria1*$^{-/-}$ or wild-type) x counterbalance (high reinforcement number cue auditory or visual; nuisance factor) x block was performed. The effect of cue was not significant, $F(1,20) = 2.64$, $p = 0.12$. There was a significant effect of block, $F(3.20,64.06) = 4.38$, $p = 0.006$, and genotype, $F(1,20) = 10.13$, $p = 0.005$. There were no significant interactions of factors, p values > 0.40. The lack of a significant difference between the reinforced cues is in contrast with the significant difference in responding to the nonreinforced cues (see below). This may suggest that the significant difference between the cues that was found with the difference scores was driven by responding to the nonreinforced cues rather than in differences in responding to the reinforced cues. It should be noted, however, that the numerical difference in responding to the high and low reinforcement number cues was greater than the difference for the nonreinforced cues. Therefore, it is likely that differences for both reinforced and nonreinforced cues contributed to the effect of cue on difference scores.

Non-reinforced cues: Gria1$^{-/-}$ mice responded to the non-reinforced cues more than wild-type mice and responding to the nonreinforced low reinforcement number cue was higher than to the

nonreinforced high reinforcement number cue for *Gria1*$^{-/-}$ mice later in training (see Figure S6b). A repeated measures ANOVA of cue (high reinforcement number or low reinforcement number) x genotype (*Gria1*$^{-/-}$ or wild-type) x counterbalance (high reinforcement number cue auditory or visual; nuisance factor) x block on responding to the non-reinforced cues showed significant main effects of cue, $F(1,20) = 8.76$, $p = 0.008$, genotype, $F(1,20) = 28.1$, $p < 0.001$, and block, $F(4.65,93.07) = 12.5$, $p < 0.001$, and significant genotype x block, $F(4.65,93.07) = 2.48$, $p = 0.041$, cue x block, $F(5.33,106.63) = 3.23$, $p = 0.008$, and cue x block x genotype, $F(5.33,106.63) = 2.49$, $p = 0.032$, interactions. The cue x genotype interaction was not significant, $F(1,20) = 2.83$, $p = 0.11$. Further analysis of the significant three-way interaction between cue, block and genotype was achieved by separate ANOVAs for each genotype. For *Gria1*$^{-/-}$ mice, there were significant main effects of cue, $F(1,10) = 6.16$, $p = 0.032$, and block, $F(4.11,41.10) = 7.68$, $p < 0.001$, and a significant cue x block interaction, $F(4.38,43.75) = 3.27$, $p = 0.02$. Further analysis of this interaction showed that there was a significant effect of cue on blocks 8, 10, and 12, F-values > 7.9, p-values < 0.02. For wild-type mice, there was a significant main effect of block, $F(3.47,34.73) = 6.86$, $p < 0.001$, but no significant main effect of cue, $F(1,10) = 3.26$, $p = 0.10$, and no significant cue x block interaction, $F(3.81,38.11) = 1.71$, $p = 0.17$.

Analysis of the temporal distribution of responding for the 40 s cue in Experiment 1

The difference in responding to the 10 s and 40 s cues in wild-type mice may reflect that responding may have come to be withheld during the early parts of the 40 s cue as training progressed. Consequently, the majority of responding may have been made closer to the time of reinforcement. This effect is known as inhibition of delay (Pavlov, 1927). In order to assess whether distribution of responding within the 40 s cue changed over the course of training we examined the raw response rates across consecutive 10 s epochs within the 40 s cue (see Figure S7). A 4 (epoch) by 12 (block) by 2 (genotype) by 2 (modality of the 40 s cue) ANOVA was performed. There was a significant effect of epoch, $F(1.28,32.92) = 34.80$, $p < 0.001$, that significantly interacted with genotype, $F(1.43, 32.92) = 3.73$, $p = 0.023$, and block, $F(6.95,159.91) = 4.52$, $p < 0.001$. The three-way interaction between genotype, epoch and block was not significant, $F(6.95,159.91) = 1.45$, $p = 0.19$. Simple main effects analysis of the genotype by epoch interaction showed that wild-type mice showed significantly lower responding for all epochs compared to *Gria1*$^{-/-}$ mice, smallest $F(1,23) = 19.47$, $p < 0.001$, 2nd epoch. Separate ANOVAs were conducted for each genotype to explore the effect of epoch for each genotype. Wild-type mice responded at a significantly lower rate in the first epoch compared to the 2nd and 3rd epochs, largest p-value = 0.049, 1st epoch versus 3rd epoch. The difference between the 1st and 4th epoch failed to reach significance, $p = 0.051$. All other comparisons were not significant, p-values > 0.99. *Gria1*$^{-/-}$ mice showed a significant effect of epoch, $F(1.24,13.58) = 27.02$, $p < 0.001$. Post-hoc comparisons using the Bonferroni correction showed that *Gria1*$^{-/-}$ mice responded at a significantly lower rate in the first epoch compared to all other epochs, largest p-value = 0.001, 1st epoch versus 4th epoch. All other comparisons were not significant, smallest p-value = 0.14, 2nd epoch versus 4th epoch. The fact that both genotypes showed a qualitatively similar effect of epoch suggests that an inhibition of delay account of reduced responding to the 40 s cue cannot explain the differences between the genotypes.

We also assessed whether the raw rates of responding during the last 10 s of the 40 s cue were lower than for 10 s cue (Figure S7). Therefore, responding was compared over equivalent time periods prior to reinforcement for each cue. If reduced responding to the 40 s cue is simply a consequence of a reduction in responding in the early portions of the 40 s cue then response rates during the last 10 s of the 40 s cue should be similar to the 10 s cue. A 2 (cue) by 12 (block) by 2 (genotype) by 2 (modality of the 10 s cue) ANOVA was performed. There was a significant effect of cue, $F(1,23) = 4.34$, $p = 0.048$, that significantly interacted with genotype, $F(1,23) = 16.00$, $p = 0.001$ and block, $F(4.73,108.78) = 2.49$, $p = 0.038$. The cue by block by genotype interaction was not significant, $F(4.73,108.78) = 2.24$, $p = 0.059$. Simple main effects analysis of the cue by genotype interaction showed that wild-type mice

responded at a significantly higher rate for the 10 s cue compared to the 40 s cue, $F(1,23) = 19.28$, $p < 0.001$, but the *Gria1*$^{-/-}$ mice did not, $F(1,23) = 1.76$, $p = 0.20$. *Gria1*$^{-/-}$ mice responded significantly more than wild-type mice for both cues, smallest $F(1,23) = 5.94$, $p = 0.023$, 10 s cue. These results demonstrate that even when responding is compared across equivalent time periods prior to reinforcement, responding was significantly lower for the 40 s cue compared to the 10 s cue for wild-type mice. Therefore, the cue duration effect in wild-type mice is not simply due to inhibition of delay.

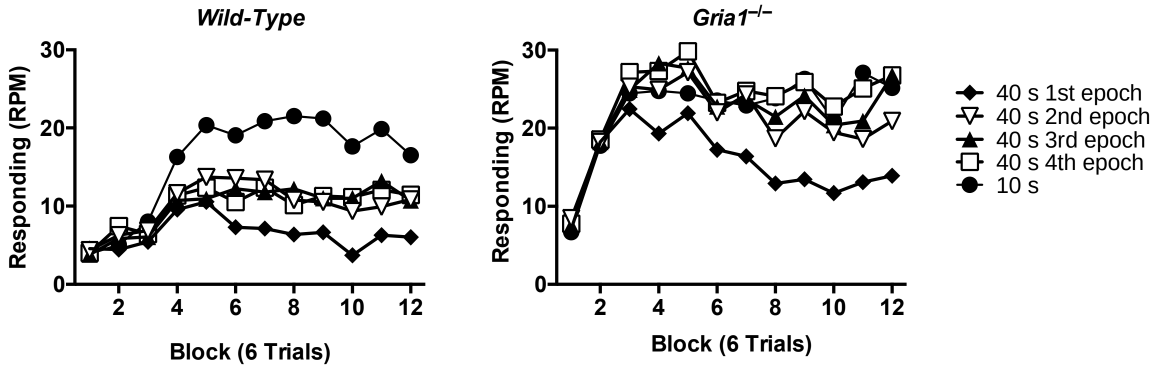

Figure S7. Mean rates of responding per minute (RPM) for the 40 s reinforced cue divided into four 10 s epochs across blocks. The rates of responding for the 10 s reinforced cue are also shown (black circles).

Analysis of Trials to Acquisition and Asymptotic Performance

Data Analysis

We initially followed the procedure used by Gallistel, Fairhurst, and Balsam (2004) of fitting cumulative Weibull functions to the response rates of mice across trials in order to determine the trial at which acquisition accorded and asymptotic levels of responding. However, meaningful parameters for acquisition and asymptote were not found for approximately a third of mice across conditions and experiments. Therefore, we instead used alternative methods described below.

Trials to acquisition: The trial at which responding was reliably above baseline for each mouse was assumed to be the point at which conditioned responding emerged. In order to determine the point at which conditioned responding emerged above baseline for each cue, we first calculated difference scores, in which pre-CS response rates were subtracted from CS response rates for each trial. The mean of the pre-CS response rates for the two reinforced cues per trial was used in order to compare the two cues to a common baseline. The cumulative difference score across trials for each cue was then calculated. By plotting the cumulative difference score it is possible to observe where responding remains at baseline level by the cumulative record being flat across trials. The point at which responding emerges above baseline results in an upward trajectory in the cumulative record across trials. We identified the point at which responding emerged above baseline by locating the trial at which there was an upward trend that was sustained over a number of trials. While this was often the point at which the cumulative difference became consistently positive, for some mice the cumulative difference score would initially decrease below zero (due to suppression of responding during the CS) before there was a positive increase. Therefore, it was necessary to determine the point at which there was an initial positive increase in the cumulative difference score rather than the point at which the score became positive. A positive slope in the cumulative difference score was determined by calculating linear trends for each consecutive block of six trials (i.e., trials 1-6, 2-7 etc.). This method was chosen rather than comparing the cumulative difference score trial by trial because it reduced the influence of trial by trial fluctuations in response rates. In order to determine if there was a

consistent positive trend in the cumulative difference score, a criterion of six consecutive trial blocks with a positive slope was used. The trial in which this condition was met was considered to be the point at which acquisition of responding occurred. For example, if the slopes for trial blocks 1-6, 2-7, 3-8, 4-9, 5-10 and 6-11 were all positive then the trials to acquisition was 11.

Asymptotic performance: Asymptotic performance for a particular cue was deemed to be the maximum response rate achieved by a mouse after acquisition of conditioned responding. From the first trial at which responding was acquired (see Trials to Acquisition analysis above) the mean response for that trial and all remaining trials was calculated. This calculation was repeated for each subsequent trial such that the mean response rates included an ever-decreasing number of trials (e.g., the mean of trials 21-72, trials 22-72, trials 23-72 etc.). However, the last mean response rate calculated was for the last remaining six trials of training in order to reduce the influence of trial by trial fluctuations in response rates on mean response rates across a small number of trials. The highest mean response rate achieved after acquisition was taken as the asymptotic level of performance.

Experiment 1: Different Reinforcement Rate - Matched Reinforcement Number

Trials to acquisition: There was little difference between genotypes and between cues (see Figure S8a). The effect of cue was not significant, $F < 1$, $p = 0.93$. The effect of genotype was not significant, $F < 1$, $p = 0.936$. There was no significant interaction of factors, $F(1,23) = 1.50$, $p = 0.23$.

Asymptote: Wild-type responded more to the 10 s cue than the 40 s cue, but this difference was smaller in *Gria1*$^{-/-}$ mice (see Figure S9a). There was a significant cue by genotype interaction, F1,23) = 4.58, $p = 0.043$. Simple main effects analysis revealed that wild-type mice responded significantly more to the 10 s cue than the 40 s cue, $F(1,23) = 26.26$, $p < 0.001$, but *Gria1*$^{-/-}$ mice did not, $F(1,23) = 3.82$, $p = 0.063$. *Gria1*$^{-/-}$ mice showed significantly greater responding to the 40 s cue than wild-type mice, $F(1,23) = 6.79$, $p = 0.016$, but this was not the case for the 10 s cue, $F < 1$, $p = 0.64$.

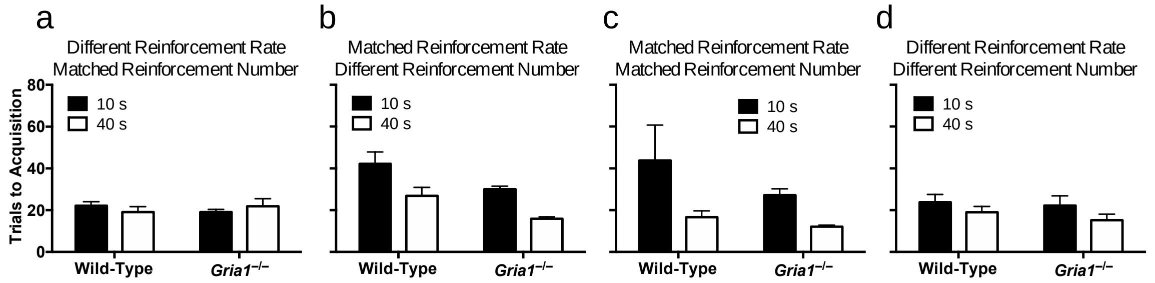

Figure S8. Mean trials to acquisition for the cue duration experiments, Experiments 1-4 in panels a-d respectively. Error bars indicate SEM.

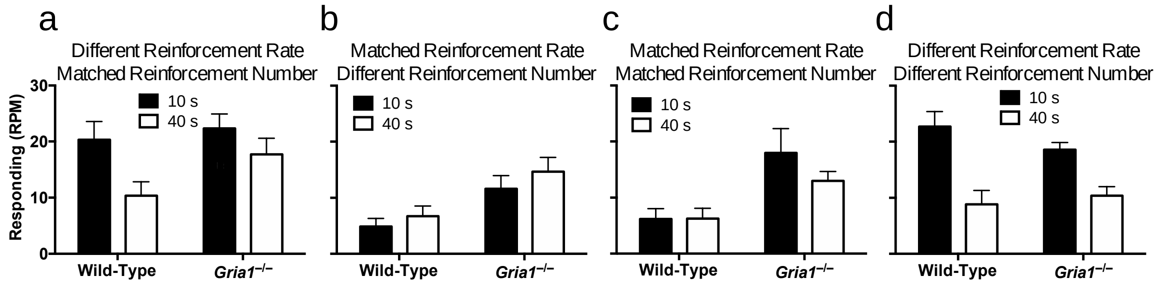

Figure S9. Mean asymptotic rates of responding for the cue duration experiments, Experiments 1-4 in panels a-d respectively. Error bars indicate SEM.

Experiment 2: Matched Reinforcement Rate - Different Reinforcement Number

Three wild-type mice failed to reach the trials to acquisition criterion for the 10 s cue. The data for these mice have been removed for both the acquisition and asymptote measures for both cues.

Trials to acquisition: Acquisition was significantly faster with the continuously reinforced 40 s cue than the partially reinforced 10 s cue, $F(1,25) = 46.84$, $p < 0.001$ (see Figure S8b). Acquisition was significantly faster for *Gria1*$^{-/-}$ mice than wild-type mice, $F(1,25) = 9.67$, $p = 0.005$. There was no significant interaction of factors, $F < 1$, $p = 0.48$.

Asymptote: There was no significant difference in responding to the reinforced cues, $F(1,25) = 2.63$, $p = 0.12$ (see Figure S9b). *Gria1*$^{-/-}$ mice responded more than wild-type mice, $F(1,25) = 12.67$, $p = 0.002$. There was no significant interaction of factors, $F < 1$), $p = 0.95$.

Experiment 3: Matched Reinforcement Rate - Matched Reinforcement Number

Trials to acquisition: Acquisition was significantly faster with the continuously reinforced 40 s cue than the partially reinforced 10 s cue, $F(1,13) = 6.34$, $p = 0.03$ (see Figure S8c). There was no significant effect of genotype, $F(1,25) = 2.01$, $p = 0.17$, or interaction of factors, $F < 1$, $p = 0.39$.

Asymptote: There was no significant effect of cue, $F(1,13)= 1.17$, $p = 0.30$ (see Figure S9c). *Gria1*$^{-/-}$ mice showed significantly greater responding than wild-type mice, $F(1,13) = 13.79$, $p = 0.003$. There was also a significant interaction of factors, $F(1,13) = 5.69$, $p = 0.03$. Simple main effects analysis showed that *Gria1*$^{-/-}$ mice showed significantly higher responding to the 10 s cue than the 40 s cue, $F(1,13) = 5.15$, $p = 0.041$, but wild-type mice did not, $F(1,13) = 1.02$, $p = 0.33$.

Experiment 4: Different Reinforcement Rate - Different Reinforcement Number

Trials to acquisition: Acquisition was significantly faster with the less frequently presented 40 s cue than the more frequently presented 10 s cue, $F(1,14) = 5.00$, $p = 0.04$ (see Figure S8d). There was no significant effect of genotype, $F < 1$, $p = 0.37$, or interaction of factors, $F < 1$, $p = 0.95$.

Asymptote: Responding was significantly greater for the 10 s cue than for the 40 s cue, $F(1,14) = 47.96$, $p < 0.001$ (see Figure S9d). There was no significant effect of genotype, $F < 1$, $p = 0.53$, or interaction of factors, $F < 1$, $p = 0.52$.

Experiment 5: Different Reinforcement Rate - Matched Reinforcement Number

Trials to acquisition: Acquisition was significantly faster with the continuously reinforced, high reinforcement rate (10 s 100%) cue than the partially reinforced, low reinforcement rate (10 s 25%) cue, $F(1,20) = 17.48$, $p < 0.001$ (see Figure S10a). There was no significant effect of genotype, $F < 1$, $p = 0.94$, or interaction of factors, $F < 1$, $p = 0.98$.

Asymptote: There was a significant cue by genotype interaction, $F(1,20) = 6.30$, $p = 0.021$ (see Figure S11a). Simple main effects analysis revealed that the interaction reflected that the effect of cue was significantly greater for the wild-type mice, $F(1,20) = 34.35$, $p < 0.001$, than the *Gria1*$^{-/-}$ mice, $F(1,20) = 7.12$, $p = 0.015$. There was no significant effect of genotype for either cue, largest $F(1,20) = 2.94$, $p = 0.10$.

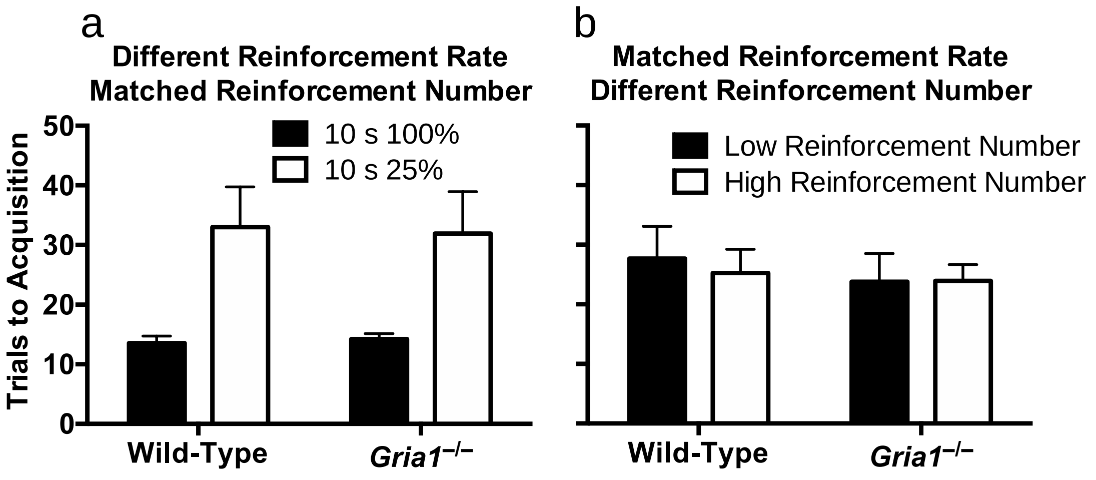

Figure S10. Mean trials to acquisition for the matched cue duration experiments, Experiments 5 and 6 in panels a-b respectively. Error bars indicate SEM.

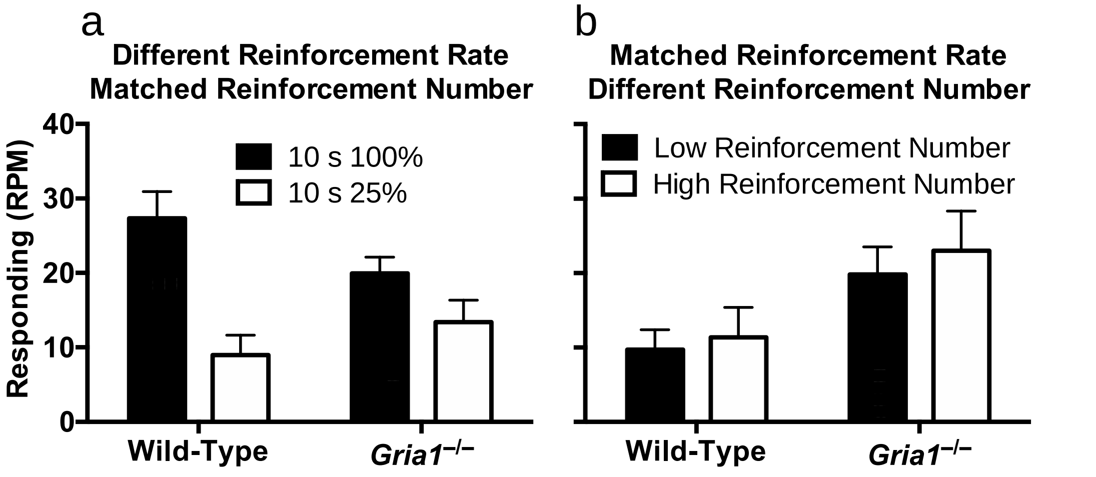

Figure S11. Mean asymptotic rates of responding for the cue duration experiments, Experiments 5 and 6 in panels a-b respectively. Error bars indicate SEM.

Experiment 6: Matched Reinforcement Rate - Different Reinforcement Number

Trials to acquisition: There was no significant effect of cue, $F < 1$, $p = 0.70$, genotype, $F < 1$, $p = 0.53$ (see Figure S10b), or interaction of factors, $F < 1$, $p = 0.68$.

Asymptote: There was no significant of cue, $F < 1$, $p = 0.38$ (see Figure S11b). *Gria1*$^{-/-}$ mice showed greater responding than wild-type mice, $F(1,20) = 6.66$, $p = 0.018$. There was no significant interaction of factors, $F < 1$, $p = 0.78$.

Timing Analysis for Experiments 1-6

Linear slopes were fitted to the normalised response rates for each mouse. For the cue duration experiments, in which mice were trained with 10 s and 40 s cues (Experiments 1-4), cue duration was normalized by comparing response rates over each tenth of the cue duration (i.e., each 1 s for the 10 s cue and each 4 s for the 40 s cue). Results were analysed using ANOVA that included the factors of cue, genotype and modality of cue (nuisance factor). The raw rates of responding within cues,

collapsed across blocks are shown in Figure S12 for the cue duration experiments (Experiments 1-4) and Figure S13 for the matched cue duration experiments (Experiments 5 and 6).

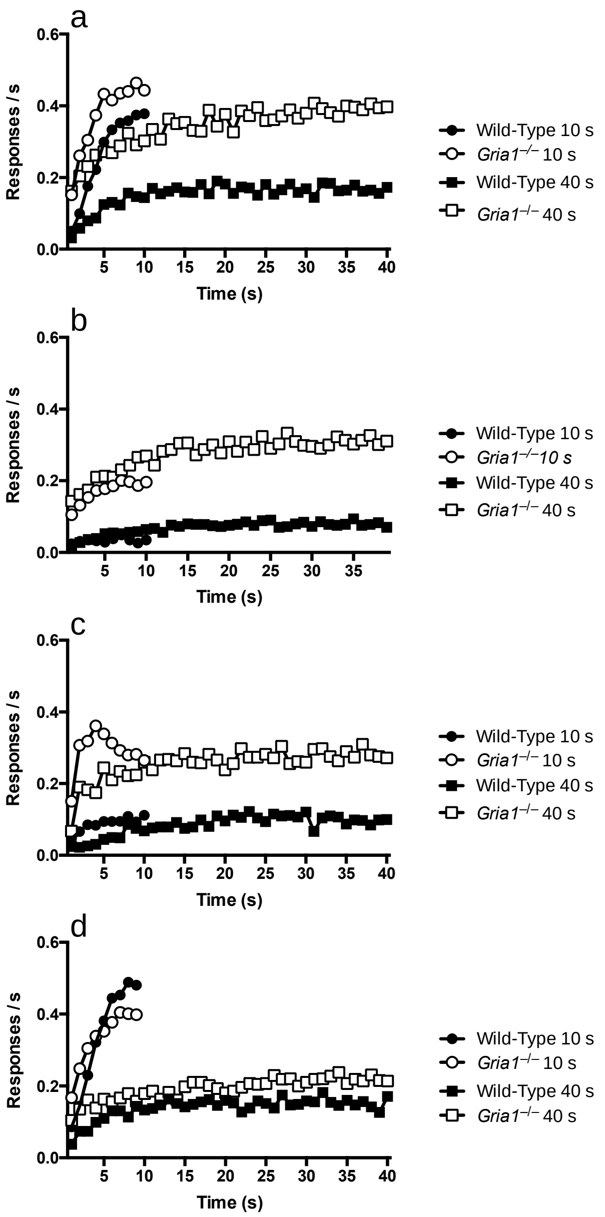

Figure S12. Mean rates of responding per second within cues for the cue duration experiments, Experiments 1-4 in panels a-d respectively. Response rates are collapsed across trials and blocks.

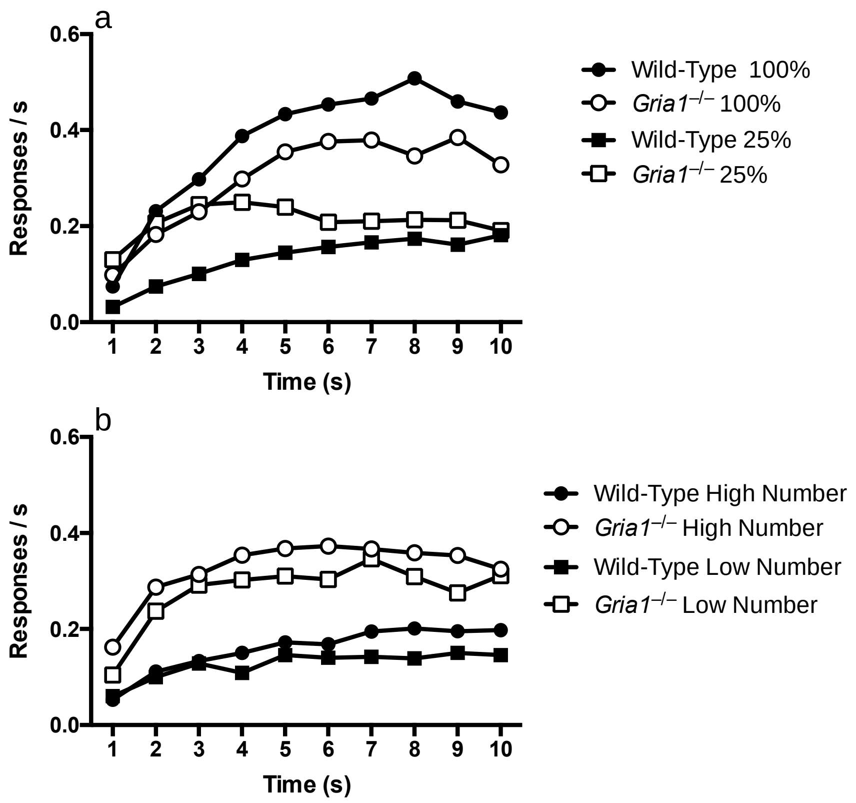

Figure S13. Mean rates of responding per second within cues for the matched cue duration experiments, Experiments 5 and 6 in panels a-b respectively. Response rates are collapsed across trials and blocks.

Experiment 1: Different Reinforcement Rate – Matched Reinforcement Number

The gradients of responding are shown in Figure S14a. There was a significant cue by genotype interaction, $F(1,23) = 8.67$, $p = 0.007$. Simple main effects analysis revealed that wild-type mice showed steeper response gradients than *Gria1*$^{-/-}$ mice for the 10 s cue, $F(1,23) = 27.16$, $p < 0.001$, but not the 40 s cue, $F(1,23) = 2.06$, $p = 0.164$. Both genotypes showed significantly steeper gradients for the 10 s cue than 40 s cue, smallest $F(1,23) = 9.37$, $p = 0.006$.

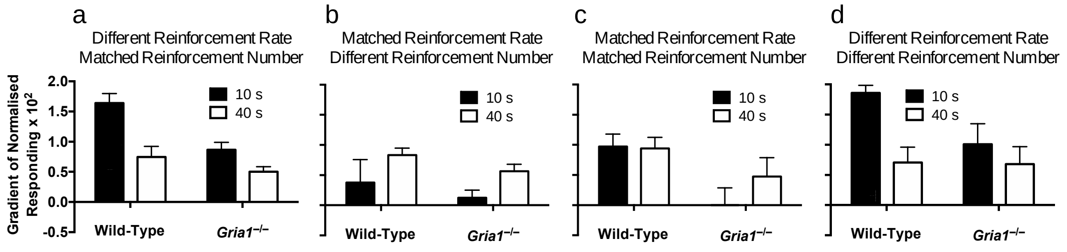

Figure S14. Mean gradients of normalised responding for the cue duration experiments (Experiments 1-4). Cue duration was normalized by comparing response rates over equivalent proportions of time within the 10 s and 40 s cues (i.e., each 1 s for the 10 s cue and 4 s for the 40 s cue). a: Different Reinforcement Rate – Matched Reinforcement Number (Experiment 1). b: Matched Reinforcement Rate – Different Reinforcement Number (Experiment 2). c: Matched Reinforcement Rate – Matched Reinforcement Number (Experiment 3). d: Different Reinforcement Rate – Different Reinforcement Number (Experiment 4). Error bars indicate SEM.

Experiment 2: Matched Reinforcement Rate – Different Reinforcement Number

The gradients of responding are shown in Figure S14b. There was no significant effect of cue duration on gradients of responding, $F(1,27) = 1.77$, $p = 0.19$, and there was no significant effect of genotype, $F < 1$, $p = 0.40$, or interaction of factors, $F(1,27) = 2.99$, $p = 0.095$.

Experiment 3: Matched Reinforcement Rate – Matched Reinforcement Number

The gradients of responding are shown in Figure S14c. Gradients of responding were significantly steeper in wild-type mice than *Gria1*$^{-/-}$ mice, $F(1,13) = 6.25$, $p = 0.027$. There was no significant effect of cue, $F(1,13) = 1.12$, $p = 0.31$, or interaction of factors, $F(1,13) = 3.39$, $p = 0.089$.

Experiment 4: Different Reinforcement Rate – Different Reinforcement Number

The gradients of responding are shown in Figure S14d. For wild-type mice, gradients of responding were significantly steeper for the 10 s cue than the 40 s cue, but this effect was less marked in *Gria1*$^{-/-}$ mice and gradients were shallower for the 10 s cue compared to wild-type mice. This was reflected by a significant cue by genotype interaction, $F(1,14) = 8.74$, $p = 0.010$. Simple main effects analysis of the interaction revealed a significant effect of genotype for the 10 s cue, $F(1,14) = 7.46$, $p = 0.016$, but not for the 40 s cue, $F < 1$, $p = 0.88$. There was an effect of cue for the wild-type mice, $F(1,14) = 35.16$, $p < 0.001$, but not for *Gria1*$^{-/-}$ mice, $F(1,14) = 1.99$, $p = 0.18$.

Experiment 5: Different Reinforcement Rate – Matched Reinforcement Number (10 s only)

The gradients of responding are shown in Figure S15a. There was no significant effect of cue, $F(1,20) = 3.78$, $p = 0.066$. Although wild-type mice showed steeper responding gradients than *Gria1*$^{-/-}$ mice, the difference was not significant, F(1,20 = 4.10, $p = 0.056$. The interaction between factors failed to reach significance, $F(1,20) = 4.286$, $p = 0.052$.

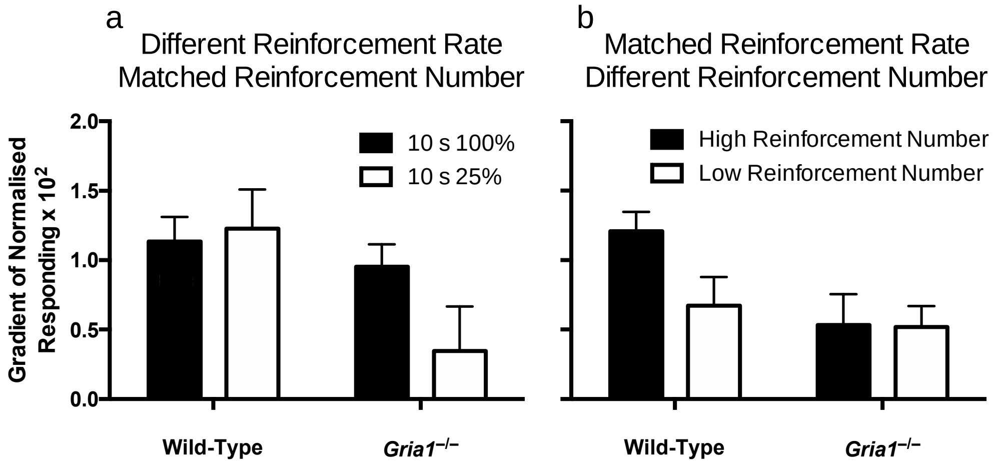

Figure S15. Mean gradients of normalised responding for the matched cue duration experiments (Experiments 5 and 6). a: Different Reinforcement Rate – Matched Reinforcement Number (Experiment 5). b: Matched Reinforcement Rate – Different Reinforcement Number (Experiment 6). Error bars indicate SEM.

Experiment 6: Matched Reinforcement Rate – Different Reinforcement Number (10 s only)

The gradients of responding are shown in Figure S15b. There was no significant effect of cue, $F(1,20) = 2.20$, $p = 0.153$. Wild-type mice showed steeper responding gradients than *Gria1*$^{-/-}$ mice, F(1,20 = 8.97, $p = 0.007$. The interaction between factors was not significant, $F(1,20) = 1.98$, $p = 0.174$.

Associative Learning Simulations

Model

Simulations of the data were conducted using an error correction rule (the Rescorla-Wagner model (Rescorla & Wagner, 1972), Eq 2) implemented in an iterative manner over time. Thus, simulation of a 40 s cue was achieved by presenting that cue four times more often than a 10 s cue, but only reinforcing one of those presentations. In this manner, the model achieves rate calculation by reducing the associative strength of the 40 s cue, compared to the 10 s cue, during those nonreinforced presentations. In this form, the model is a version of temporal difference learning (Sutton & Barto, 1990), in which the discount factor equals zero.

$$\Delta V_A = [\alpha_A \beta](\lambda - V_A) \qquad \text{Eq. 2}$$

In this model, the change in associative strength for cue A on a given trial, ΔV_A, is calculated by multiplying the product of the CS learning rate parameter, α_A, and the US learning rate parameter, β, by the discrepancy between maximal US conditioning, λ, and the current associative strength of cue A, V_A. The US learning rate parameter can take different values for excitatory, β_+, and inhibitory, β_-, learning during reinforced and nonreinforced trials, respectively.

All simulations were performed using the Rescorla & Wagner Simulator (Version 4) published by Mondragón, Alonso, Fernández, and Gray (2012). The numbers of presentations of each cue were consistent with those in the corresponding experimental data, with the distinction mentioned previously that 40 s cues were presented as though they consisted of four 10 s cues, only one of which was reinforced. For simulation of the wild-type data, the CS learning rate parameter for all cues was set to 0.2, the excitatory and inhibitory US learning rate parameters were set to 0.5, and the maximal US conditioning was set to be 1 during reinforced trials and 0 during nonreinforced trials. For simulation of the *Gria1*$^{-/-}$ data, the inhibitory US learning rate parameter was set to 0, to impair the decrements in associative strength as a result of nonreinforcement, with all other parameters remaining the same as for wild-type mice.

Cue Duration Experiments – Experiment 1-4

Wild-type mice show learning that is dependent on reinforcement rate, whereas *Gria1*$^{-/-}$ mice respond to cues based on their reinforcement number (Figure 1a-h, main manuscript). Simulations of these experiments accurately model the behaviour of both wild-type and *Gria1*$^{-/-}$ mice (Figure S16a-h). For the *different reinforcement rate – different reinforcement number* experiment, simulation of the *Gria1*$^{-/-}$ manipulation shows a benefit for the 10 s cue over the 40 s cue early in training, which disappears as learning to both cues reaches asymptotic levels (Figure S16g-h). This is somewhat at odds with the behaviour of the *Gria1*$^{-/-}$ mice, which show a benefit for the 10 s cue over the 40 s cue throughout training (Figure 1g-h, main manuscript). It is possible that this is a result of the model reaching asymptotic levels of conditioning before the *Gria1*$^{-/-}$ mice. As such, with extended training, the *Gria1*$^{-/-}$ mice might be expected to reach similar asymptotic performance for both the 10 s and 40 s cues, in accordance with the simulation.

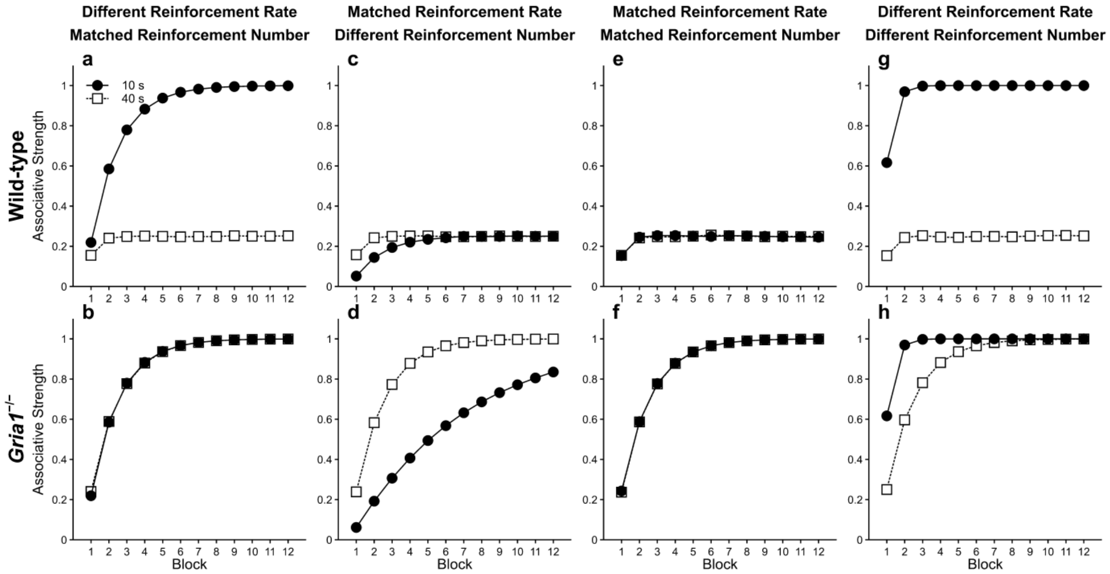

Figure S16. An error correction rule applied iteratively over time closely simulates the reinforcement rate-dependent behaviour of wild-type mice in four cue duration experiments. Reducing the inhibitory US learning rate parameter mimics the pattern of responding in Gria1$^{-/-}$ mice with responding being dependent on the number of reinforcements rather than reinforcement rate. Panels a and b: cues differed in reinforcement rate, but not reinforcement number. Panels c and d: cues were matched for reinforcement rate, but not reinforcement number. Panels e and f: cues were matched for reinforcement rate and reinforcement number. Panels g and h: cues differed in reinforcement rate and reinforcement number. Simulation data should be compared with the corresponding panels in Figure 1 (main manuscript).

Matched Cue Duration Experiments – Experiment 5-6

When cue durations are matched, wild-type mice show sensitivity to reinforcement rate and reinforcement number, whereas *Gria1*$^{-/-}$ mice show impaired sensitivity to reinforcement rate (Figure 3a-d, main manuscript). Simulations of these experiments model the behaviour of wild-type and *Gria1*$^{-/-}$ mice quite successfully (Figure S17a-d). In the *different reinforcement rate – matched reinforcement number* experiment (Experiment 5), *Gria1*$^{-/-}$ mice show a numerical, but not statistically significant, benefit for the 10 s 100% cue over the 10 s 25% cue late in training (Figure 3b, main manuscript) that is not captured by the model (Figure S17b). This may be the result of noise in the behavioural data or a failure of the model. In the *matched reinforcement rate – different reinforcement number* experiment, wild-type mice showed a benefit for the high reinforcement number cue over the low reinforcement number cue (Figure 3c, main manuscript) that was only evident in the model early in training (Figure S17c). As discussed previously, this could be the result of the model reaching asymptotic levels of conditioning before the wild-type mice.

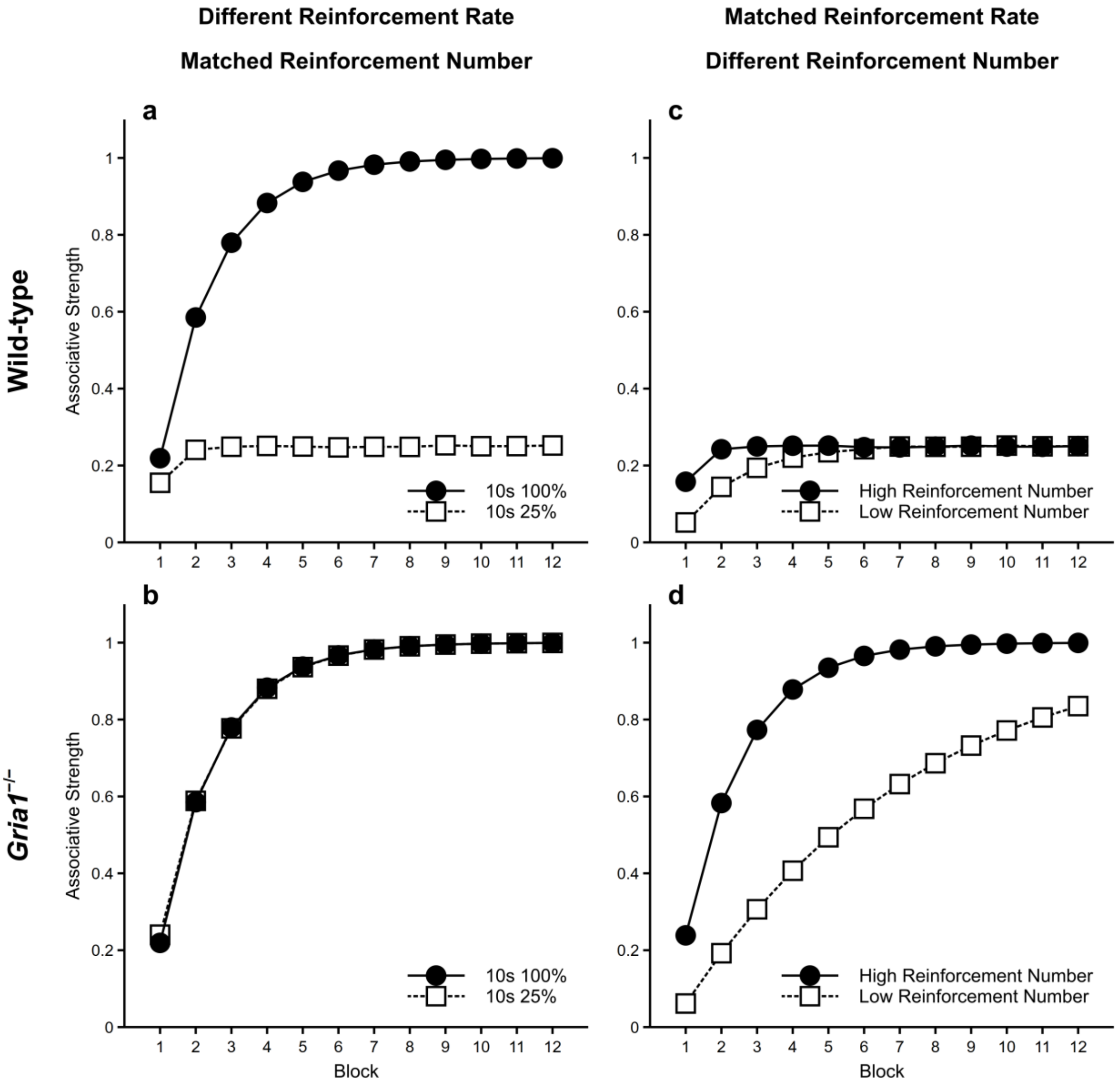

Figure S17. An error correction rule applied iteratively over time closely simulates the reinforcement rate-dependent behaviour of wild-type mice in two matched cue duration experiments. Reducing the inhibitory US learning rate parameter mimics the Gria1$^{-/-}$ mice being dependent on the number of reinforcements rather than reinforcement rate. Panels a and b: cues differed in reinforcement rate, but not reinforcement number. Panels c and d: cues were matched for reinforcement rate, but not reinforcement number. Simulation data should be compared with the corresponding panels in Figure 3 (main manuscript).

Extinction – Experiment 7

Both wild-type and *Gria1*$^{-/-}$ mice showed similar levels of extinction to a nonreinforced cue that had previously been reinforced (Figure S1a-b). While a simulation of this experiment accurately models the behaviour of wild-type mice (Figure S18a), modelling the behaviour of *Gria1*$^{-/-}$ mice by reducing the inhibitory learning rate parameter impairs extinction, such that the associative strength of the newly nonreinforced cue remains high (Figure S18b). This is clearly at odds with the behaviour of the *Gria1*$^{-/-}$ mice (Figure S1b), suggesting that their dependence on the number of reinforcements rather than the reinforcement rate cannot be modelled, in all circumstances, by assuming a reduced inhibitory learning rate parameter.

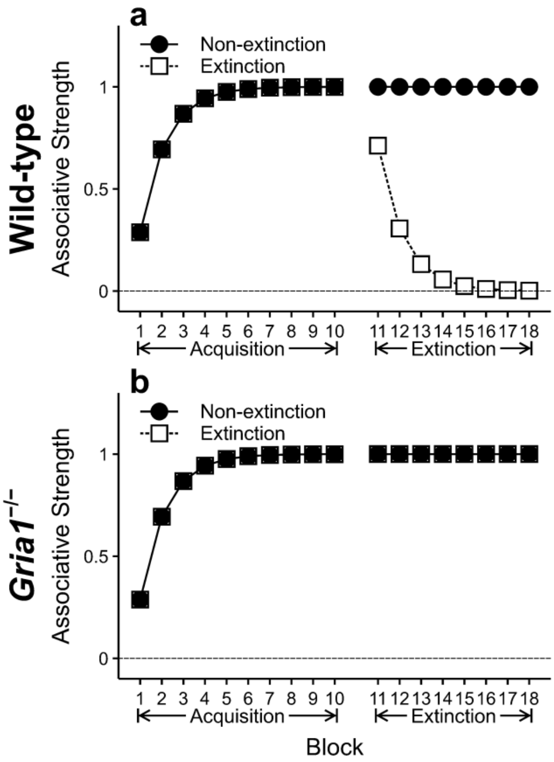

Figure S18. Panel a: an error correction rule applied iteratively over time closely simulates the behaviour of wild-type mice in the extinction experiment. Panel b: reducing the inhibitory US learning rate parameter predicts an absence of extinction, but this was not consistent with the behaviour of Gria1$^{-/-}$ mice. Simulation data should be compared with the corresponding panels in Figure S1.

Supplementary References

Balsam, P. D., & Gallistel, C. R. (2009). Temporal maps and informativeness in associative learning. *Trends Neurosci, 32*(2), 73-78.

Gallistel, C. R., Fairhurst, S., & Balsam, P. (2004). The learning curve: implications of a quantitative analysis. *Proc Natl Acad Sci U S A, 101*(36), 13124-13131.

Gallistel, C. R., & Gibbon, J. (2000). Time, rate, and conditioning. *Psychological Review, 107*(2), 289-344.

Kvalseth, T. O. (1985). Note on R^2. *The American Statistician, 39*, 279=285.

Mondragón, E., Alonso, E., Fernández, A., & Gray, J. (2012). Rescorla & Wagner Simulator (Version 4). St. Albans, UK: CAL-R.

Pavlov, I. P. (1927). *Conditioned Reflexes*. London: Oxford University Press.

Rescorla, R. A., & Wagner, A. R. (1972). A theory of Pavlovian conditioning: variations in the effectiveness of reinforcement and nonreinforcement. In A. H. Black & W. F. Prokasy (Eds.), *Classical conditioning, II: Current research and theory* (pp. 64-99). New York: Appleton-Century-Crofts.

Schmajuk, N. A., Gray, J. A., & Lam, Y. W. (1996). Latent inhibition: a neural network approach. *J Exp Psychol Anim Behav Process, 22*(3), 321-349.

Sutton, R. S., & Barto, A. G. (1981). Toward a modern theory of adaptive networks: expectation and prediction. *Psychol Rev, 88*(2), 135-170.

Sutton, R. S., & Barto, A. G. (1990). Time derivative models of Pavlovian reinforcement. In M. Gabriel & J. Moore (Eds.), *Foundations of Adaptive Networks* (pp. 539-602). Cambridge, MA: MIT Press.

Tam, S. K. E., Jennings, D. J., & Bonardi, C. (2015). Effects of Dorsal Hippocampal Damage on Conditioning and Conditioned-Response Timing: A Pooled Analysis. *Hippocampus, 25*(4), 444-459.

Wagner, A. R. (1981). SOP: A model of automatic memory processing in animal behavior. In N. E. Spear & R. R. Miller (Eds.), *Information processing in animals: Memory mechanisms* (pp. 5-47). Hillsdale, NJ: Lawrence Erlbaum Associates Inc.
